# A prospective observational study of plasma concentrations and safety of combined intravenous lidocaine and epidural ropivacaine in laparotomy surgery

**DOI:** 10.1371/journal.pone.0344277

**Published:** 2026-03-06

**Authors:** Ottilie Trocheris-Fumery, Alexandre Pruvot, Paul Tarpin, Sophie Collette, Sandra Bodeau, Momar Diouf, Jean-Marc Régimbeau, Hervé Dupont, Stéphane Bar, Youssef Bennis, Osama Abou-Arab

**Affiliations:** 1 Department of Anesthesiology and Critical Care Medicine, Amiens Picardy University Hospital, Amiens, France; 2 Centre for pain assessment and treatment, Amiens Picardy University Hospital, Amiens, France; 3 Department of Clinical Pharmacology, Amiens Medical Centre, Amiens, France; 4 Clinical research division, Amiens Hospital University, Amiens, France; 5 Department of Digestive Surgery, Amiens Picardy University Hospital, 1, rondpoint du Pr-Cabrol, Simplifying Care for Complex Patients, UR-UPJV SSPC, Clinical Research Unit, University of Picardie Jules-Verne, Amiens, France; IRCCS: IRCCS Ospedale San Raffaele, ITALY

## Abstract

Intravenous lidocaine (IVL), an alternative to epidural analgesia (EA) for laparotomies, has demonstrated anti-inflammatory and anti-hyperalgesic effects. However, its combination with EA raises concerns about systemic toxicity. This study aimed to assess the safety of this combination in patients undergoing laparotomy. We conducted a prospective observational study at Amiens University Hospital in France, involving adult patients scheduled for laparotomies. Intravenous lidocaine was administered at induction as a 1.5 mg/kg bolus (ideal body weight), followed by a continuous infusion of 2 mg/kg/h maintained until the end of surgery. An epidural catheter was inserted before surgery, prior to induction of general anesthesia, no local anesthetic was administered intraoperatively. Epidural analgesia was initiated at the end of surgery with a 5 mg bolus of ropivacaine, followed by a continuous infusion. The primary outcome was the occurrence of lidocaine and ropivacaine plasma concentrations outside established safety ranges. Lidocaine plasma concentrations were measured 30 minutes after bolus (LP1), at the end of surgery (LP2), and two hours after discontinuation of the infusion (LP3). Ropivacaine concentrations were measured two hours (RP1) and 24 hours after initiation of epidural infusion (RP2). The secondary endpoint the assessment of cardiac and neurological toxicity within 48 hours post-surgery. Fifty patients were included from March 2022 to March 2024. One patient exceeded the lidocaine threshold (LP2), and another exceeded the ropivacaine threshold (RP2). LP1 was 1.8 [1.5–2.4] µg/ml, LP2 was 2.1 [1.6–2.4] µg/ml, and LP3 was 1.2 [0.8–1.7] µg/ml. Ropivacaine plasma concentration was 126 [87–211] µg/L at RP1, and 785 [524–1318] µg/L at RP2. No cardiac or neurological adverse events were observed. The combination of EA and IVL appeared to be safe for major abdominal surgery. Clinical trials Number NCT05368753. EudraCT Number: 2021-005508-37.

## Introduction

The multifactorial mechanisms underlying postoperative pain are increasingly well understood. [1] With growing evidence linking inflammation and both central and peripheral sensitization to pain, some researchers have aimed to optimize analgesic protocols to mitigate surgery-induced inflammation. [2]

Epidural analgesia (EA) is the gold standard for major abdominal surgery via laparotomy, with robust evidence supporting its efficacy in acute postoperative pain relief from large randomized trials. [3] However, there is no conclusive evidence demonstrating additional benefits for chronic pain relief and its anti-inflammatory effects remain debated. [4,5] Part of this uncertainty stems from technical complications and challenges in Enhanced Recovery After Surgery (ERAS) protocols, prompting some teams to explore alternatives such as intravenous lidocaine (IVL).

IVL has been shown to significantly reduce acute postoperative pain, opioid consumption, and inflammation. [6] Increasing evidence suggests that lidocaine may also help manage chronic postoperative pain. A recent meta-analysis found that lidocaine was associated with significant reductions in pro-inflammatory markers. [7] Its effects appear most pronounced when administered intraoperatively, with benefits lasting days to weeks, suggesting a potential role in preventing central or peripheral nervous system sensibilization. [8,9] However, a Cochrane review found weaker evidence for perioperative lidocaine infusions in major abdominal surgery compared with previous reports. [10]

While studies have compared epidural analgesia (EA) and intravenous lidocaine (IVL) individually, research on the potential benefits of their combination remains limited. This combined approach may provide complementary effects on postoperative pain control, inflammation, and recovery. However, it also raises important safety considerations, particularly regarding the risk of local anesthetic systemic toxicity (LAST), a rare but serious complication. In 2021, British experts issued warnings about the inappropriate use of lidocaine and provided guidelines for safe practice. However, these recommendations are based on expert opinion due to limited data regarding the combination of ropivacaine and lidocaine. [11]

The primary objective of this study was to evaluate the safety of combining EA and IVL and to explore its potential clinical benefits for future research. Specifically, we conducted a feasibility study to measure plasma local anesthetic concentrations in patients undergoing major abdominal surgery who received IVL followed immediately by EA ropivacaine infusion.

## Method

### Ethics

We conducted a prospective observational single-centre study at Amiens University Hospital from March 2022 to March 2024. Ethical approval for this study (reference 2021−086 B) was provided by the Ethics Committee “CPP Sud Est III” on 9 November 2021. The trial was registered before patient enrolment on ClinicalTrials.gov (identifier number: NCT05368753) and in EudraCT (identifier number: 2021-005508-37). The recruitment period for this study ran from March 9, 2022 to March 27, 2024. Written informed consent was obtained from all study participants before inclusion according to French guidelines on clinical research. [12] The study was reported in accordance with the STROBE guidelines for observational studies. [13]

### Population study

The population study was patient undergoing major abdominal surgery with laparotomy, age ≥ 18 years and informed written consent. Exclusion criteria were major laparoscopic surgery, contraindications to thoracic epidural analgesia (patient refusal, allergy to ropivacaine, localized infection at the puncture site or documented systemic infection, hemostasis disorders, Prothrombin time (PT) < 70% or platelet count < 70 000 mm^-3^), contraindications to IVL (allergy, unpaced high-degree atrioventricular conduction block, uncontrolled epilepsy, estimated glomerular filtration rate < 30 ml/min/m² and not on dialysis, hepatic insufficiency, porphyria, concomitant use of antiarrhythmic drugs associated with torsade de pointes (amiodarone, disopyramide, quinidine, sotalol)), patients under guardianship or curatorship or deprived of liberty, and cognitive impairment incompatible with obtaining informed consent. Simple arrhythmias (e.g., extrasystoles or atrial fibrillation) were not considered contraindications to intravenous lidocaine unless patients were receiving antiarrhythmics from the same electrophysiological class.

### Study protocol

**Fig 1:**
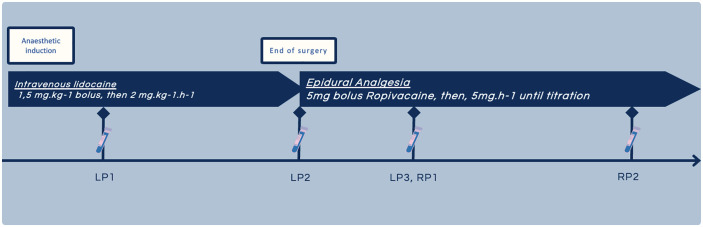
Protocol study, LP1: lidocaine concentration 30 minutes after the bolus, LP2: lidocaine concentration at the end of the infusion, LP3: lidocaine concentration two hours after the start of the epidural ropivacaine injection. RP1: ropivacaine concentration two hours after the start of the epidural and RP2: ropivacaine concentration twenty-four hours after the start of the epidural..

Patients received IVL in the intra operative time followed by an EA in the postoperative time once the surgery ended and after the interruption of IVL. The study protocol was summarized in Fig 1.

#### Management of IVL.

A lidocaine bolus was administered at a dose of 1.5 mg.kg^-1^ of ideal body weight at induction, followed by an infusion using a syringe pump at a dose of 2 mg.kg^-1^.h^-1^ of ideal body weight (Fig 1).

The medication used was Xylocard® 50 mg/ml, diluted in saline solution to a concentration of 20 mg/ml. This infusion was stopped at the end of the surgery. Patients had blood samples taken to measure lidocaine levels 30 minutes after the bolus (LP1) to measure lidocaine plasma concentration post-distribution, at the end of the infusion (LP2) to capture steady-state plasma levels, and two hours after the start of the epidural ropivacaine injection (LP3) following the cessation of intravenous lidocaine infusion – to assess lidocaine plasma concentration after approximately one elimination half-life. [14]

#### Management of EA.

An epidural catheter was placed at the beginning of the procedure, prior to general anesthesia.

No local anesthetic was injected through the catheter during surgery. At the end of the surgery, EA was initiated, with a bolus of 5 ml of ropivacaine at a concentration of 1 mg/ml, followed by a continuous infusion starting at 5 mg.h^-1^. The infusion rate could subsequently be adjusted according to the patient’s level of analgesia and the results of cold–warm sensory testing performed in the postoperative period. Blood samples were collected two hours (RP1) and twenty-four hours (RP2) after the initiation of the epidural infusion to assess ropivacaine plasma concentrations following the absorption phase and close to steady state, respectively. [15,16]

#### Anesthesia management.

General anesthesia was conducted according to guideline with sufentanil, hypnotic, and curare. The choice of hypnotic ad curare drug is at the discretion of the physician. Before induction, preoxygenation is carried out using a facial mask until an end tidal oxygen fraction of 90% is reached. Dexamethasone (8 mg) and ketamine (0.5 mg/kg) were administered at induction.[17] After orotracheal intubation, mechanical ventilation was started with a tidal volume of 8 ml/kg ideal body weight, a positive end expiratory pressure of 5 cmH_2_O a respiratory rate to obtain an end tidal CO_2_ between 35 and 40 mmHg, and an oxygen fraction to target oxygen saturation>95%. The depth of anesthesia was monitored with a bispectral index target between 40 and 60. Pain multimodal management with paracetamol, nefopam, non-steroidal anti-inflammatory drug (according to the patient’s contraindications) and opioid for uncontrolled pain (NRS ≥ 6 immediately or > 3 despite previous use of other analgesics). [17] An electrocardiogram was performed in the post-anesthesia care unit and compared to the reference (prior to anesthesia).

### Endpoints

The primary outcome measure was the occurrence of plasma concentrations of lidocaine and ropivacaine outside the safe range at different monitoring times.

For lidocaine, the therapeutic plasmatic range was defined between 1.4 and 5 µg/ml. [18] Neurological toxicity occurred around a plasmatic concentration of lidocaine over 5 µg/ml. [6] Over 6 µg/ml, patients experienced trouble in speeches (slurred speech and tinnitus). Over 10 µg/ml, patients experienced loss of conscious. Cardiac toxicity occurred over a plasmatic concentration of 10 µg/mL (15–20 µg/mL). [19]

For ropivacaine, according to one previous report, we defined the threshold for the therapeutic plasmatic range under 2200 µg/L. [20]

The secondary outcome measure was the occurrence of cardiac or neurological toxicity within the 48 hours after surgery, including the following events: central nervous system disturbances such as seizures, dizziness, paraesthesia, tongue numbness, hyperacusis, tinnitus, visual disturbances, dysarthria, muscle contractions, tremors, hypoesthesia; and cardiac disturbances such as bradycardia, tachycardia, conduction disorders potentially leading to cardiac arrest

### Follow up

Patients were followed for 48 hours after the end of surgery. Pain was monitored one a day for 48 hours using the numerical rating scale (NRS) at rest after the surgery. Data related to the epidural (infusion rate, effectiveness via hot/cold test) were collected. Neurological monitoring was performed every 8 hours during the infusions. A standardized clinical checklist, based on the SFAR (Société Française d’Anesthésie-Réanimation) guideline for local anesthetic systemic toxicity, was used to screen for early neurological or cardiovascular signs of toxicity.

All evaluators had access to this official reference sheet approved by the SFAR, ensuring consistent assessment and reporting of any suspected adverse effects.

### Lidocaine and ropivacaine plasma concentration monitoring

Blood was centrifuged for 15 min at 1100 g within 2 h of sampling, and the plasma was stored at −80°C until assayed. Lidocaine and ropivacaine plasma concentrations were measured by reverse-phase liquid chromatography coupled with tandem mass spectrometry detection operated in the positive ion mode. Validation of the procedure was conducted in accordance with guidelines on bioanalytical methods of the European Medicines Agency [21].

The calibration model was linear over the range of 0.23–6.7 µg/mL for lidocaine and 0.5–500 µg/L for ropivacaine. Intra-assay and inter-assay bias and coefficient of variation were within ± 20% for all analytes at the lower limit of quantification (0.23 µg/ml for lidocaine, 0.5 µg/L for ropivacaine) and within ±15% at remaining concentrations.

### Sample size

The sample size was informed by published perioperative studies reporting widely variable incidences of plasma lidocaine or ropivacaine concentrations exceeding safety thresholds. [22–25] Assuming that approximately 10% of patients might fall outside the predefined safety range, the inclusion of 50 patients allowed us to estimate this proportion with a 95% confidence interval of acceptable width (± 8%)

### Statistical analysis

Data were presented as median [interquartile range] or as number (percentage), as appropriate.

Primary endpoint was the rate of measurements outside of the safety range and was expressed as the number (percentage) of values over the upper limit of the predefined safety range (5 µg/ml for lidocaine and 2200 µg/L for ropivacaine)

Comparisons of repeated plasma lidocaine and ropivacaine over time was proceeded using a Kruskal-Wallis test given the absence of assumption for the variance homogeneity (Levene test) and the non-normal distribution of the residual (Shapiro test). Tests for assumptions were available in Supporting Information. (S1 Fig, S2 Fig, S3 Fig, S4 Fig).

Multivariate linear regression analysis was performed to evaluate the effects of age, body mass index, gender type, ß blocker medication, albumin, hepatic and renal functions on the plasmatic concentrations of lidocaine at LP and ropivacaine at RP. The ß coefficients for each factor from the linear regression, along with their 95% confidence intervals (95% CI), were reported for each variable. The accuracy of the model was assessed with the report of the R^2^ and the P value for the model. Assumptions for the use of a multivariate linear regression (normal distribution of residuals, absence of collinearity, homogeneity of variance, were assessed and reported in a supplemental file. Statistical analyses were conducted using R Studio software (Version 2024.12.0 + 467). A P-value <0.05 was considered statistically significant.

## Results

### Population study

**Fig 2:**
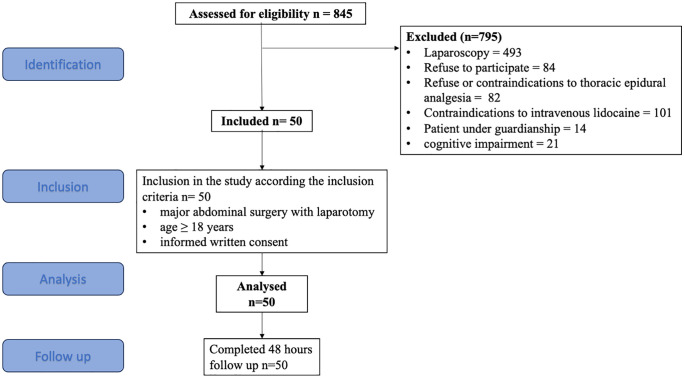
Flow chart.

From March 2022 to March 2024, 845 patients were eligible, 50 were included from march 2022 to march 2024 and analyzed as reported in the flow chart of the study (Fig 2).

Briefly, the demographic and per operative characteristics of the patients are detailed in Table 1. The median age of the cohort was 69 years [61−75]. There was a majority of men (55%) and the median BMI was 24.6 kg m^-2^ [22.1; 27.9]. All patients except 2 went for cancer surgery. 6% of patients had chronic kidney disease. The median prothrombin level was 93% [86;100]. Digestive surgery was the most represented (75%) followed by urology then gynecology. 20% of patients had a chronic medication with ß blocker.

**Table 1:** Demographic and per operative characteristic, results presented in median and percentage.

Variables	n = 50
Age; *years*	69 [61-75]
Female gender; (n) *%*	22 (45)
BMI, *kg/m2*	24.6 [22.1; 27.9]
Prothrombin level, *%*	93% [86;100]
Preoperative albumin; *mg/L*	39.1 [35.7; 41−5]
Medical history; n (*%)*	
Hypertension	22 (43)
Diabetes	7 (14)
ß-blocker medication	10 (20)
Cancer	48 (96)
Chronic kidney disease	8 (16)
ASA; *%* II III	58 42
Renal Clearance; *ml/min/m2*	88 [70 −105]
Surgery duration; *minutes*	300 [210-409]
*Type of surgery; n (%)*	
Urology	7 (14)
Cystoprostactectomy Nephrectomy	6 1
Digestive Pancreatic Surgery Colorectal surgery Hepatic surgery Gastric surgery	37 (74) 20 11 3 3
Gynaecology (Debulking Ovarian surgery)	6 (12)
*Post operative stay; n (%)*	
Surgical ward recovery	10
ICU recovery	90

**ASA:** American Status Anaesthesia**; BMI:** body mass index**; ICU:** intensive care unit, Renal clearance expressed as MDRD estimation

### Intraoperative management

The median duration of surgery was at 300 [210; 409] minutes. Intraoperative blood loss was estimated at 300 [125; 600] ml. Intraoperative fluid replacement was 3000 mL [2000; 4000]. Anesthetic induction was performed with propofol at a median dose of 2.2 [1.9; 2.5] mg.kg^-1^ in 96% of cases. The other drug used was etomidate at a median dose of 0.4 mg.kg^-1^. Ketamine was used in 94% of cases at a median dose of 0.5 [0.4–0.53] mg.kg^-1^. The main neuromuscular blockade drug was represented by rocuronium for 94% of cases (median dose at 0.7 mg.kg^-1^ [0.6–0.8]). Sufentanil was used in 71% of cases (0.32 [0.26;0.38] µg/kg). In other cases, the opioid used was remifentanil. The anesthesia was maintained with halogenated agents (sevoflurane).

### Primary endpoints

Two patients had concentrations over the toxic thresholds. One patient (2%) had an LP2 level of 5.2 µg/mL and one other (2%) patient had an RP2 level of 2540 µg/L.

**Fig 3:**
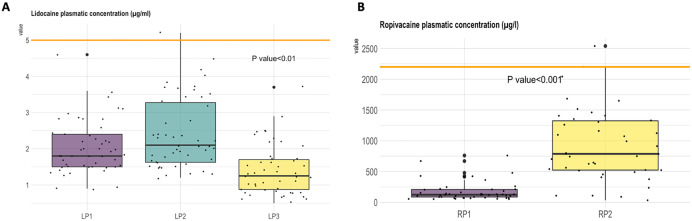
A. Dot plot of lidocaine plasmatic level measured 30 minutes after the bolus (LP1), at the end of surgery (LP2), and two hours after the end of the infusion (LP3). Ropivacaine plasmatic concentration was measured at two hours. **3B** Dot plot of ropivacaine plasmatic level at 2 hours (**RP1**) and 24 hours (**RP2**) after the start of the continuous infusion.

The lidocaine plasmatic concentration at LP1 was at 1.8 [1.5; 2.4] µg/mL. The plasma concentration at LP2 was at two hours after the bolus and continuous infusion (LP2) was 2.1 [1.6; 3.3] µg/mL. The plasmatic concentration at LP3 was at 1.2 [0.8; 1.7] µg/mL (Fig 3A). The ropivacaine plasma concentration at RP1 was at 126 [87; 210] µg/L. The ropivacaine plasmatic concentration at RP2 was at 785 [524; 1318] µg/L (Fig 3B).

### Secondary endpoint

No patient in the study showed signs of local anesthetic toxicity. No post-operative electrocardiogram performed immediately after surgery showed any changes. Two patients experienced complete arrhythmias due to atrial fibrillation on post-operative days 1 and 2, both returning to sinus rhythm by the third day. The analysis of the electrocardiograms is detailed in Table 2.

**Table 2:** Primary and secondary outcomes, results presented in median and percentage.

Variables	n = 50	P value
Lidocaine plasmatic level (µg/mL)		
LP1	1.8 [1.5 - 2.4]	–
LP2	2.1 [1.6 - 3.2]	–
LP3	1.2 [0.8 - 1.7]	–
Ropivacaine plasmatic level (µg/L)		
RP1	126 [7 - 210]	–
RP2	785 [524 - 1318]	–
Signs of LAST (%)		
Neurological	0 (0)	–
Cardiac	0 (0)	–
Electrocardiogram		
PR (ms)		
*Baseline*	156 [138-172]	0.195
*After surgery*	161 [141-176]	
QRS (ms)		
*Baseline*	95 [87-104]	0.283
*After surgery*	95 [87-103]	
QTc (ms)		
*Baseline*	434 [417-443]	0.219
*After surgery*	441 [420-450]	
HR (bpm)		
*Baseline*	72 [65-82]	**0.049**
*After surgery*	77 [70-88]	

**LAST**: local anaesthetic systemic toxicity; **ms**: milliseconds; **bpm**: beats per minute; Lidocaine plasmatic level 30 minutes after bolus (**LP1**), at the end of the surgery (**LP2**) et 2 hours after the end of the surgery (**LP3**); Ropivacaine plasmatic level two hours after the bolus in the epidural (**RP1**) et 24 hours after (**RP2**).

### Local anesthetics data and factor

The local anesthetic data are detailed in Table 2. The median intravenous lidocaine dose administered to patients was at 1.5 [1.4; 1.6] mg.kg^-1^ of ideal body weight for the bolus, followed by 2.0 [1.9; 2.1] mg.kg^-1^.h^-1^ of ideal body weight for the continuous infusion.

Five patients received an additional bolus within the hour following the end of the surgery due to an inadequate metameric level or lack of analgesia. The median infusion rate of ropivacain over the following 48 hours was 8 [7;10] ml. h^-1^. The median duration of epidural maintenance was 48 [32; 61] hours (EA details available in Supporting information, S1 Table).

**Fig 4:**
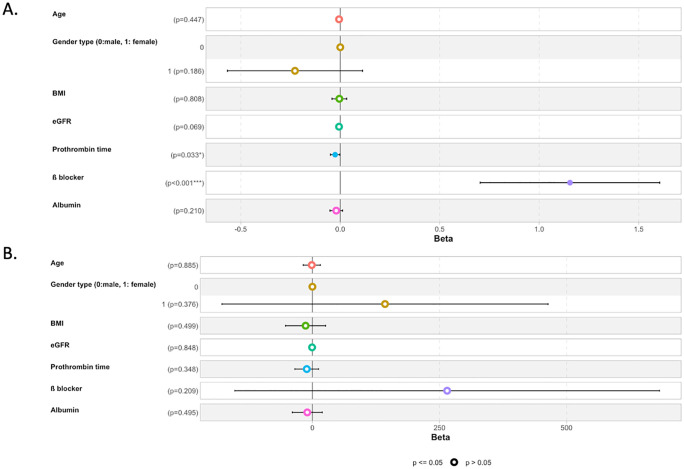
Forest plots of multivariate linear regression assessing factors that might influence plasmatic concentration of lidocaine and ropivacaine. ß coefficient for each factor and its confidence interval is represented. **BMI:** body mass index; **eGFR**: glomerular filtration rate.

Lidocaine or ropivacaine plasmatic concentration was not significantly influenced by either age, gender type or BMI (Fig 4).

The multivariate regression model including age, gender type, BMI, glomerular filtration rate, Prothrombin time, B blocker medication and albumin (R^2^ = 30%). In multivariable regression analysis, β-blocker use showed an influence on plasma lidocaine concentration following intravenous administration (β = 1.02; 95%CI p < 0.001). Prothrombin time also had an influence on plasma lidocaine concentration, (β = 0,3; 95% CI; p = 0.033).

Age, sex, body mass index, estimated glomerular filtration rate, and serum albumin did not influence plasma lidocaine concentration after multivariable adjustment. Estimated glomerular filtration rate showed a non-significant trend toward an association

The multivariate regression model including age, gender type, BMI, glomerular filtration rate, Prothrombin time, ß blocker medication and albumin. did not influence ropivacaine plasma concentrations (R^2^ = 8%).

### Pain assessment

The median value of the NRS, at rest, assessed two hours after surgery was at 3 [0; 4]; at 24 hours it was 1 [1; 2] and at 48 hours it was 1 [0; 2].

## Discussion

This is the first study to evaluate the plasma concentrations of two local anesthetics used concomitantly in the same patient. In our cohort, this combination was not associated with a systematic increase in plasma concentrations nor with clinical signs of local anesthetic toxicity. At the same time, patients did not experience clinically significant pain according to the observed pain intensity scores.

The IVL concentrations observed in our study were consistent with those reported in the literature [23,26] and the observed variability in measurements aligns with findings from other studies. [22] This interindividual variability may be explained by well-known pharmacokinetic determinants such as differences in body weight and body composition, liver function and hepatic blood flow, cardiac output, protein binding capacity, and perioperative physiological changes, all of which can substantially influence plasma concentrations. We chose to use ideal body weight for all lidocaine administration, ensuring safety, as only one patient slightly exceeded the toxic threshold. By adhering to the 2021 UK recommendations [11], we demonstrated the safe plasma use of lidocaine. However, in our cohort, 16% of patients had LP1 levels below the therapeutic threshold of 1.5 µg/mL, and 10% had LP2 levels below the efficacy threshold despite adherence to protocol. These findings raise the hypothesis that using ideal body weight for lidocaine dosing, although safe, may not achieve optimal therapeutic concentrations in all patients. This could partly explain the lack of efficacy reported by Paterson et al. in a recent study [27], which also applied ideal body weight–based dosing and capped continuous infusions at 120 mg/h [28] Future studies should investigate alternative strategies, potentially incorporating lean body mass or adjusted body weight, to better balance efficacy and safety.

There is ongoing debate about dose adjustments for obese patients due to the observed increase in volume of distribution and subsequent prolongation of half-life (t1⁄2). [29] Standard dosing based on total or ideal body weight may not suit these patients.[14] Some authors suggest using actual body weight for bolus dosing and ideal body weight for continuous infusion, while others advocate for adjusted weight for BMI > 30 kg/m². [18,30] In our study, one patient reached the safety threshold for plasma lidocaine concentration (LP2, 5.2 µg/mL) despite dosing adjusted to ideal body weight. This 73-year-old woman (65 kg, eGFR 40 mL/min/1.73 m²) was receiving long-term nebivolol therapy and underwent a nephroureterectomy lasting 229 minutes. Her ropivacaine plasma concentration also reached 2043 µg/L, although the epidural infusion rate (7 mg/h) fully complied with the study protocol. No neurological or cardiovascular toxicity was observed. While neither condition alone contraindicates lidocaine, their combination may affect its pharmacokinetics. Beta-blockers and lidocaine compete for α₁- acid glycoprotein (AAG) binding, potentially increasing the free fraction of lidocaine. Consistently, multivariable regression analysis showed that β-blocker medication modulate plasmatic lidocaine concentration. Pharmacokinetic studies recommend dosage adjustments for renal clearance below 30 ml/min/1.73 m², highlighting gaps in our understanding of lidocaine pharmacology, particularly in patients with renal impairment. [14] These findings emphasize the importance of recognizing relative contraindications and other factors influencing lidocaine accumulation, which are not fully addressed in current guidelines.

We observed substantial inter-individual variability in ropivacaine plasma concentrations, particularly at the RP2 time point, consistent with previous reports. [31] Such variability may result from differences in catheter positioning and from clinical adjustments in bolus dosing or continuous epidural infusion rates during hospitalization. Although total dose and infusion rate contribute to systemic exposure, pharmacokinetic studies show that plasma concentrations are largely determined by epidural absorption kinetics, tissue distribution, and patient-specific factors. [15,16,32,33] Despite uncertainty in epidural catheter positioning, which may have contributed to variability in plasma local anesthetic concentrations, the majority of concentrations remained below toxicity thresholds, and no local anesthetic systemic toxicity or related adverse events were observed.

In our study, we did not report high toxic plasma levels, although such events remain theoretically possible the metabolism of lidocaine and ropivacaine. Both lidocaine and ropivacaine share a common hepatic metabolic pathway, specifically the cytochrome P450 (CYP) family, CYP3A4 and CYP1A2, suggesting potential competition in their metabolism when used concomitantly. [14,34]. One plausible explanation for the absence of elevated levels is that lidocaine and ropivacaine were sampled at different phases of their systemic exposure lidocaine during its elimination phase and ropivacaine during its absorption phase. Moreover, the slow absorption of ropivacaine from the epidural space into the plasma provides an additional safety margin. [31,35]. In addition to assessing plasma concentrations, we focused on monitoring cardiac and neurologic effects. Electrocardiographic monitoring in the post-anesthesia care unit and hospital wards revealed no changes in QRS or PR intervals.

Several limitations of this study must be acknowledged. First, the sample size (n = 50), although adequate for pharmacokinetic description, is insufficient to draw definitive conclusions about the incidence of local anesthetic systemic toxicity (LAST), which remains a rare event. Second, this was a single-center descriptive cohort without a control group, which limits the generalizability of the findings to other patient populations, surgical settings, or anesthetic practices.

Third, neurological and cardiovascular safety assessments were based on standardized clinical monitoring rather than formal neurocognitive or electrophysiological testing, which may have limited the sensitivity for detecting subclinical or asymptomatic conduction disturbances. In addition, variability in surgical duration, infusion times, and individual patient factors (such as age, body composition, renal and hepatic function, or albumin level) could have influenced plasma concentrations. Moreover, while sparse sampling reduces constraints, it may not fully characterize the drugs’ pharmacokinetic profile, and thus cannot definitively rule out toxic concentrations during the entire follow-up period. In addition, the absence of free (unbound) plasma concentration measurements precludes a precise evaluation of the pharmacologically active fraction of ropivacaine and lidocaine.

Future studies should measure the free fraction of ropivacaine to provide additional insights. In one patient with marked hypoalbuminemia, ropivacaine plasma concentration (RP2) exceeded the commonly reported toxicity threshold (2540 µg/L; > 2200 µg/L), although no clinical signs of systemic toxicity were observed. This 64-year-old woman (BMI 16 kg/m², eGFR 90 mL/min/1.73 m², serum albumin 27 g/L) underwent a pelvectomy lasting 480 minutes. She received standard intravenous lidocaine dosing and a continuous epidural ropivacaine infusion at 12 mg/h for 28 hours, in full compliance with the study protocol. Increased perioperative AAG levels may have elevated total drug concentrations without affecting the free fraction. [36] These findings underscore the importance of individualized pharmacokinetics and vigilance for potential drug interactions. We believe that the dosing strategy should rely on a personalized approach, integrating patient-specific demographic and nutritional characteristics, as well as the type of surgery and its potential hemodynamic impact. Such an individualized framework would better account for the complex interactions that influence lidocaine pharmacokinetics in the perioperative setting, and further research is needed to develop and validate such strategies.

The latest British guidelines [11] recommend maintaining a minimum interval of four hours between the discontinuation of intravenous lidocaine and the administration of an epidural local anesthetic bolus. This conservative recommendation may, however, conflict with enhanced recovery after surgery (ERAS) protocols, which emphasize early multimodal analgesia and rapid postoperative recovery.

In our study, epidural ropivacaine was initiated immediately after discontinuation of intravenous lidocaine, and no clinical or biological signs of local anesthetic systemic toxicity were observed.

Although these results are preliminary and based on a limited sample, they challenge the current 4-hour interval recommendation and suggest that a shorter transition period could be safe under close monitoring and standardized dosing conditions. Further large-scale, controlled pharmacokinetic studies are warranted to confirm these findings and to help inform potential updates to existing safety guidelines.

In our study, the concomitant use of EA with ropivacaine and IVL did not result in increased plasma concentrations of local anesthetics or signs of local anesthetic toxicity. These findings support further investigation of the potential clinical benefits of this combined approach.

## Supporting information

S1 Table Characteristics of local anesthetic use, results presented in median and percentage. IQR: interquartile range.
(DOCX)

S1 Fig Assumptions for using the ANOVA test for lidocaine plasma concentrations. (DOCX)

S2 Fig Assumptions for using the ANOVA test for ropivacaine plasma concentrations. (DOCX)

S3 Fig Assumptions for using multivariable linear model for lidocaine plasma concentrations. (DOCX)

S4 Fig Assumptions for using multivariable linear model for ropivacaine plasma concentrations. (DOCX)

S1 File Consort check list. (DOCX)

S2 File Study protocol. (DOCX)
