# Supplementary material for: A prospective observational study of plasma concentrations and safety of combined intravenous lidocaine and epidural ropivacaine in laparotomy surgery
Source: PLoS One. 2026 Mar 6;21(3):e0344277. doi: 10.1371/journal.pone.0344277 (PMC12965542; doi:10.1371/journal.pone.0344277)
Supplement: S1 Table — IQR: interquartile range. (DOCX) [file pone.0344277.s001.docx]

**S1 Table. Characteristics of local anesthetic use, results presented in median and percentage. IQR:** interquartile range

| **Variables** | **n = 50** |
| --- | --- |
| *Intravenous lidocaine dose (ideal body weight)* |  |
| Bolus, mg.kg^-1^, [IQR] | 1.48 [1.36; 1.53] |
| Continuous infusion, mg.kg-1.h-1, [IQR] | 2.0 [1.9; 2.1] |
| *Epidural Follow - up* |  |
| Epidural infusion, mL/h, median [IQR] | 8 [7; 10] |
| Time of epidural infusion, hours, median [IQR] | 48 [32; 61] |
| Epidural efficient (%) | 84 |
| Epidural removed before 48h (%) | 4 |
| Lateralized epidural (%) | 8 |
| Epidural repositioned (%) | 4 |
