# Supplementary figures and images for: A prospective observational study of plasma concentrations and safety of combined intravenous lidocaine and epidural ropivacaine in laparotomy surgery
Source: PLoS One. 2026 Mar 6;21(3):e0344277. doi: 10.1371/journal.pone.0344277 (PMC12965542; doi:10.1371/journal.pone.0344277)

**S1 Figure. Assumptions for using the ANOVA test for lidocaine plasma concentrations**


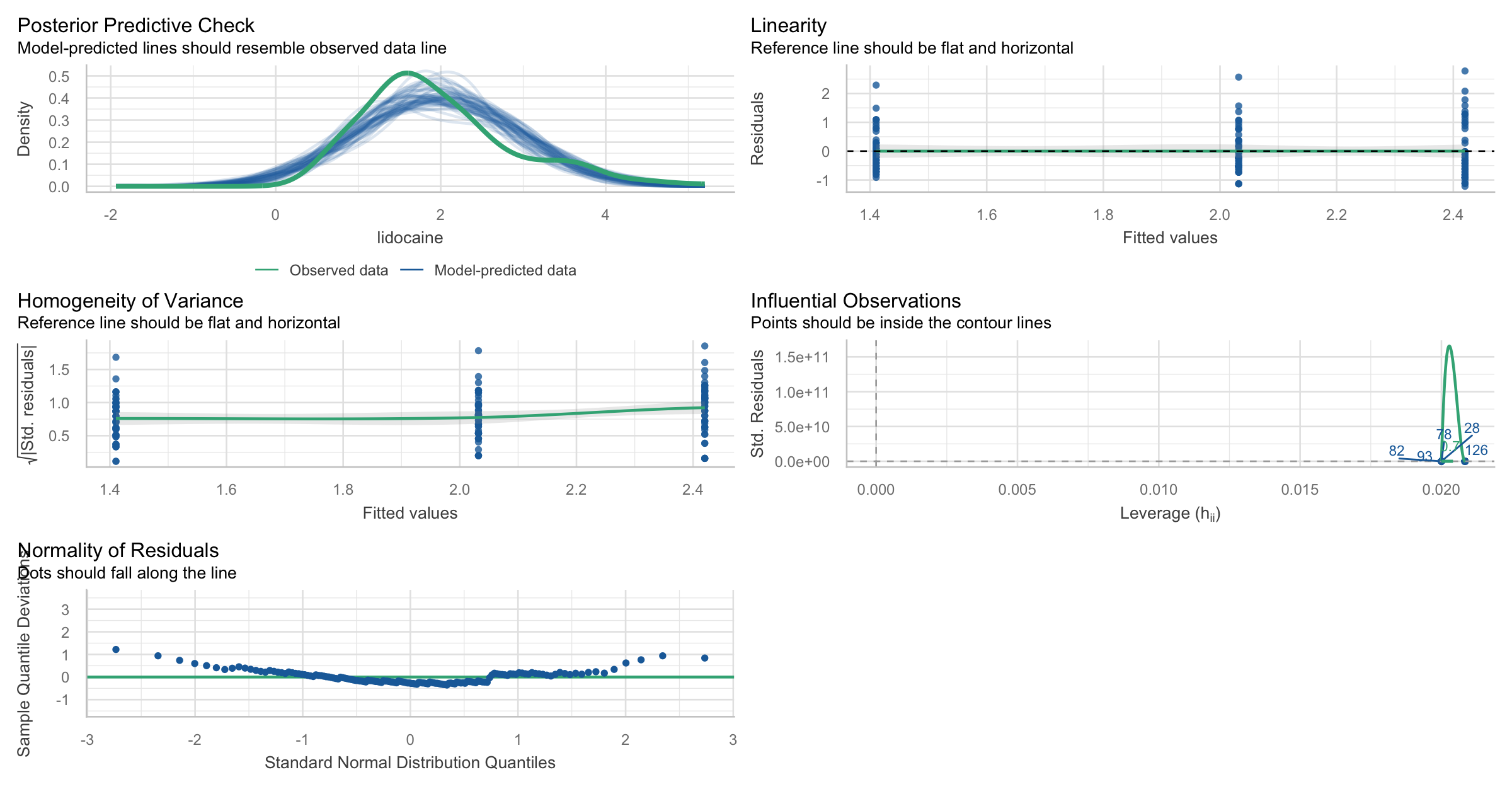

Supplement: S1 Fig — (DOCX) [file pone.0344277.s002.docx]

**S2 Figure. Assumptions for using the ANOVA test for ropivacaine plasma concentrations**


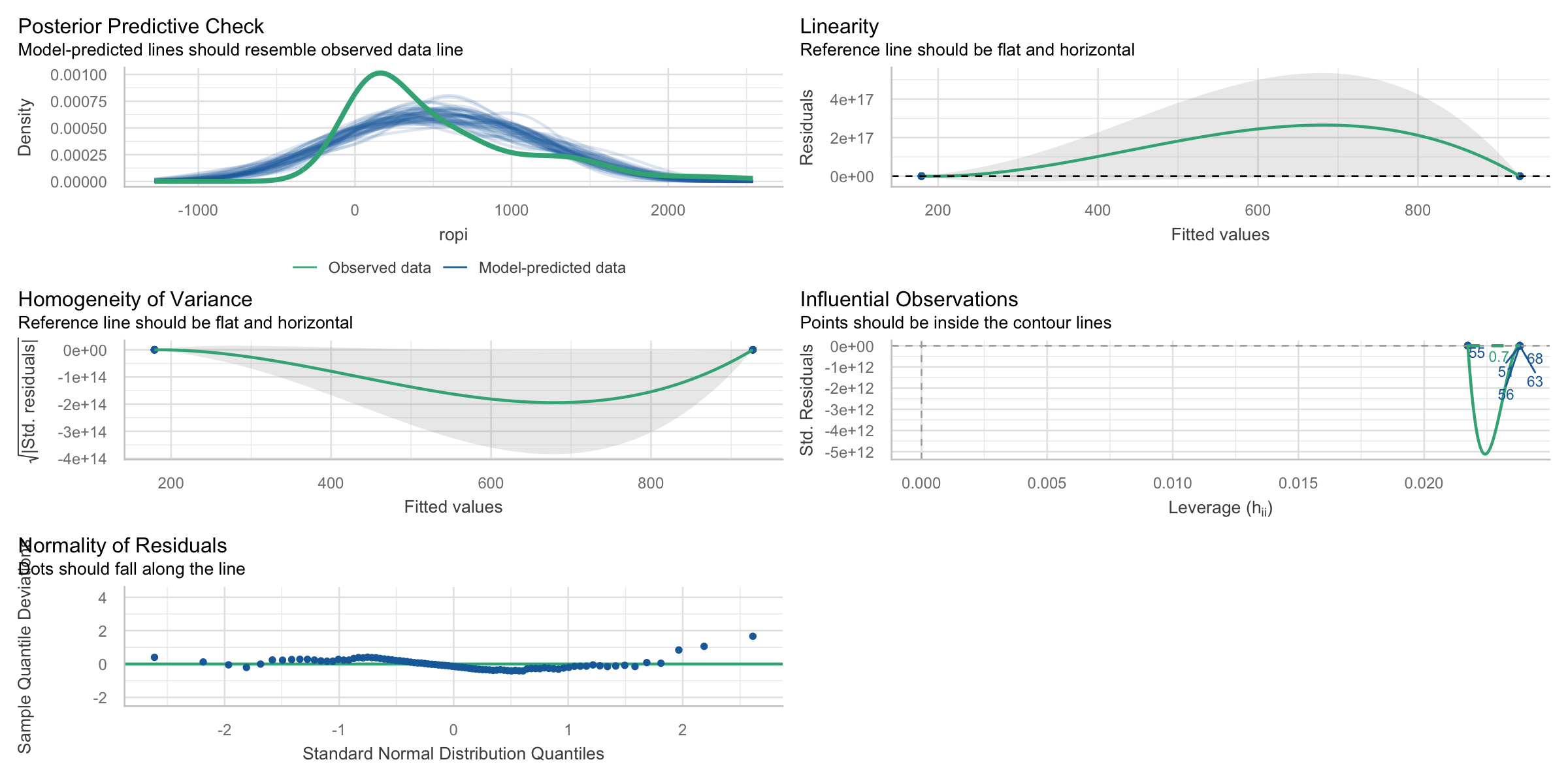

Supplement: S2 Fig — (DOCX) [file pone.0344277.s003.docx]

**Consort Check List**

**
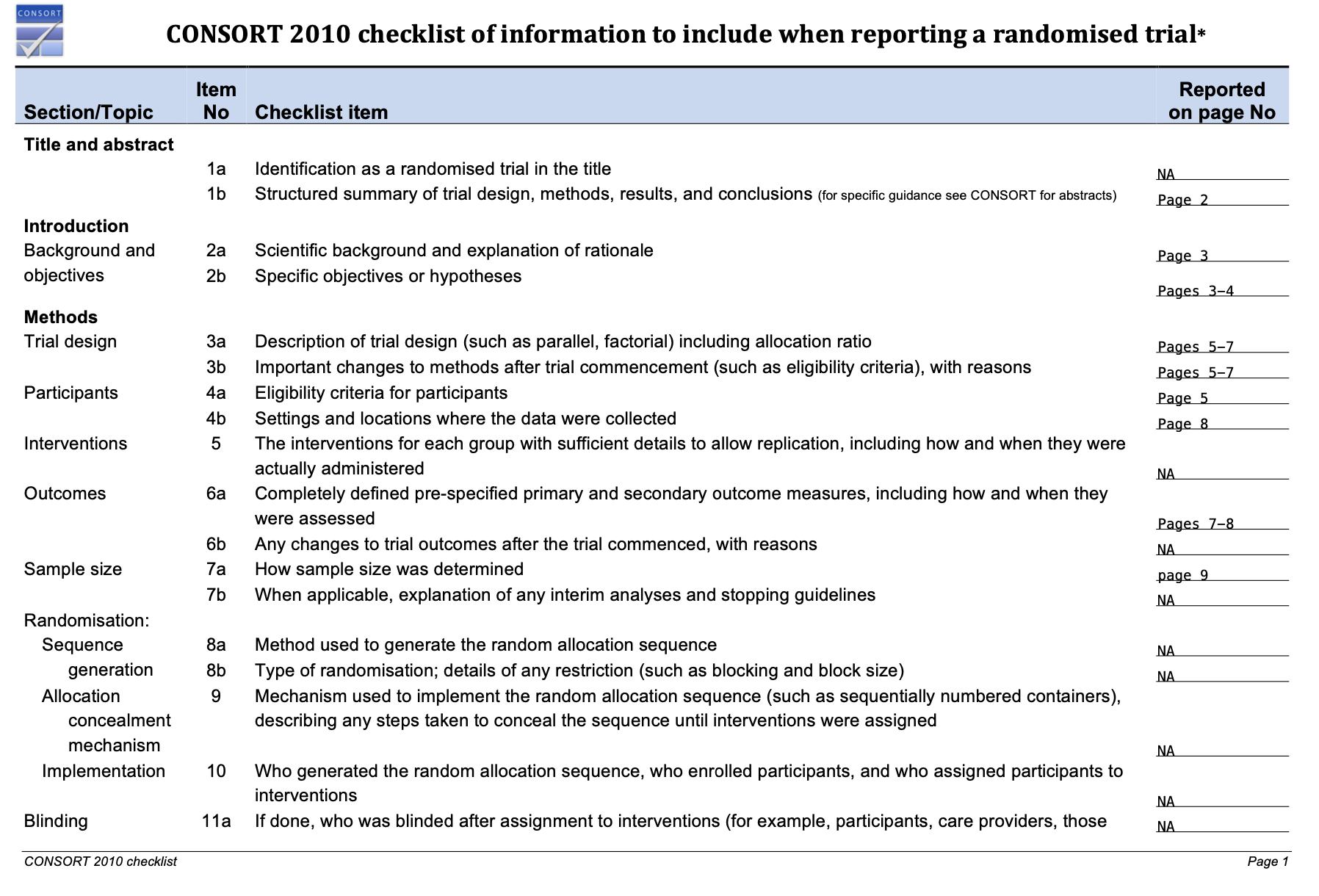

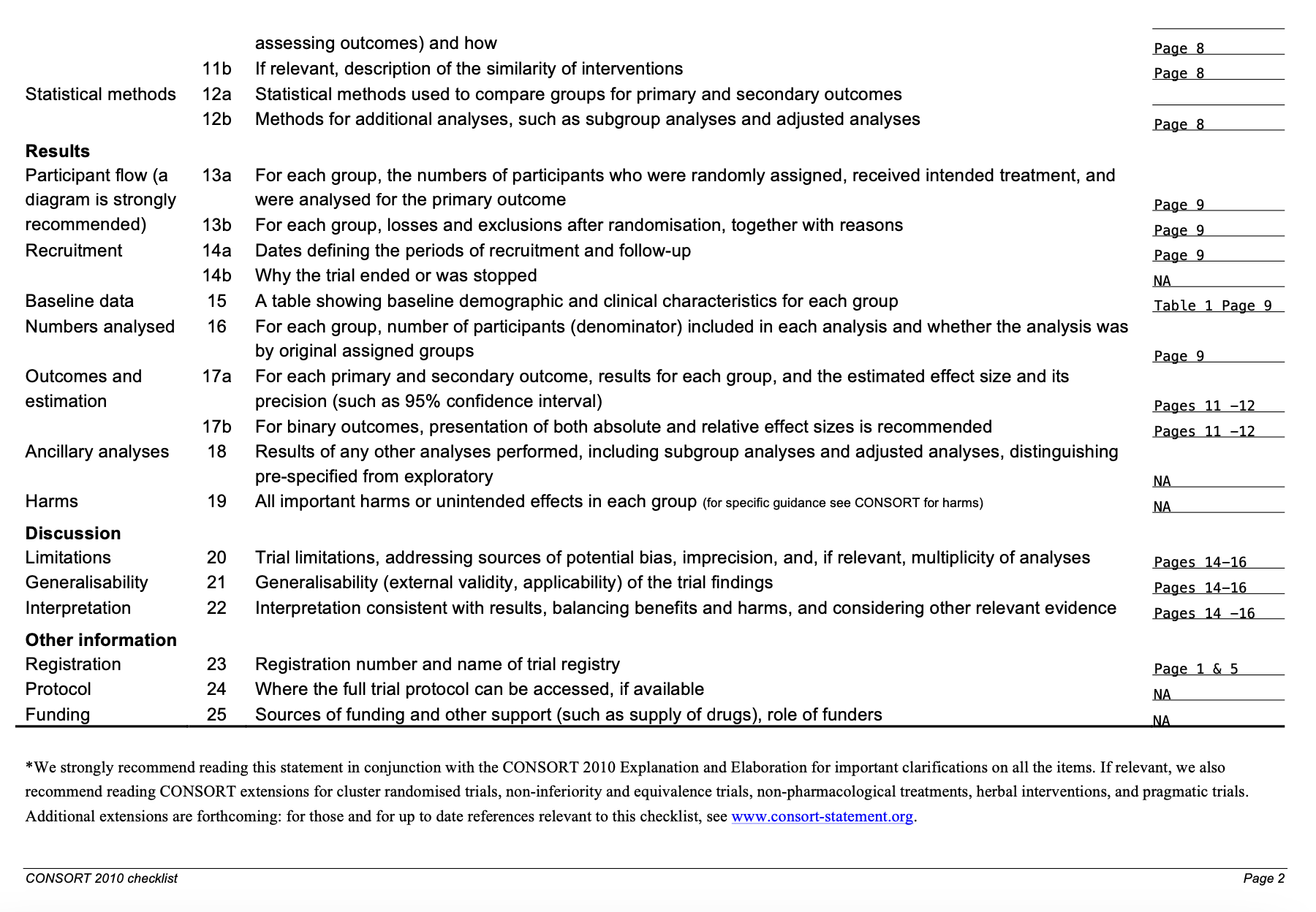
**

Supplement: S1 File — (DOCX) [file pone.0344277.s006.docx]
