# Supplementary material for: A prospective observational study of plasma concentrations and safety of combined intravenous lidocaine and epidural ropivacaine in laparotomy surgery
Source: PLoS One. 2026 Mar 6;21(3):e0344277. doi: 10.1371/journal.pone.0344277 (PMC12965542; doi:10.1371/journal.pone.0344277)
Supplement: S4 Fig — (DOCX) [file pone.0344277.s005.docx]

**S4 Figure. Assumptions for using multivariable linear model for ropivacaine plasma concentrations.**

**
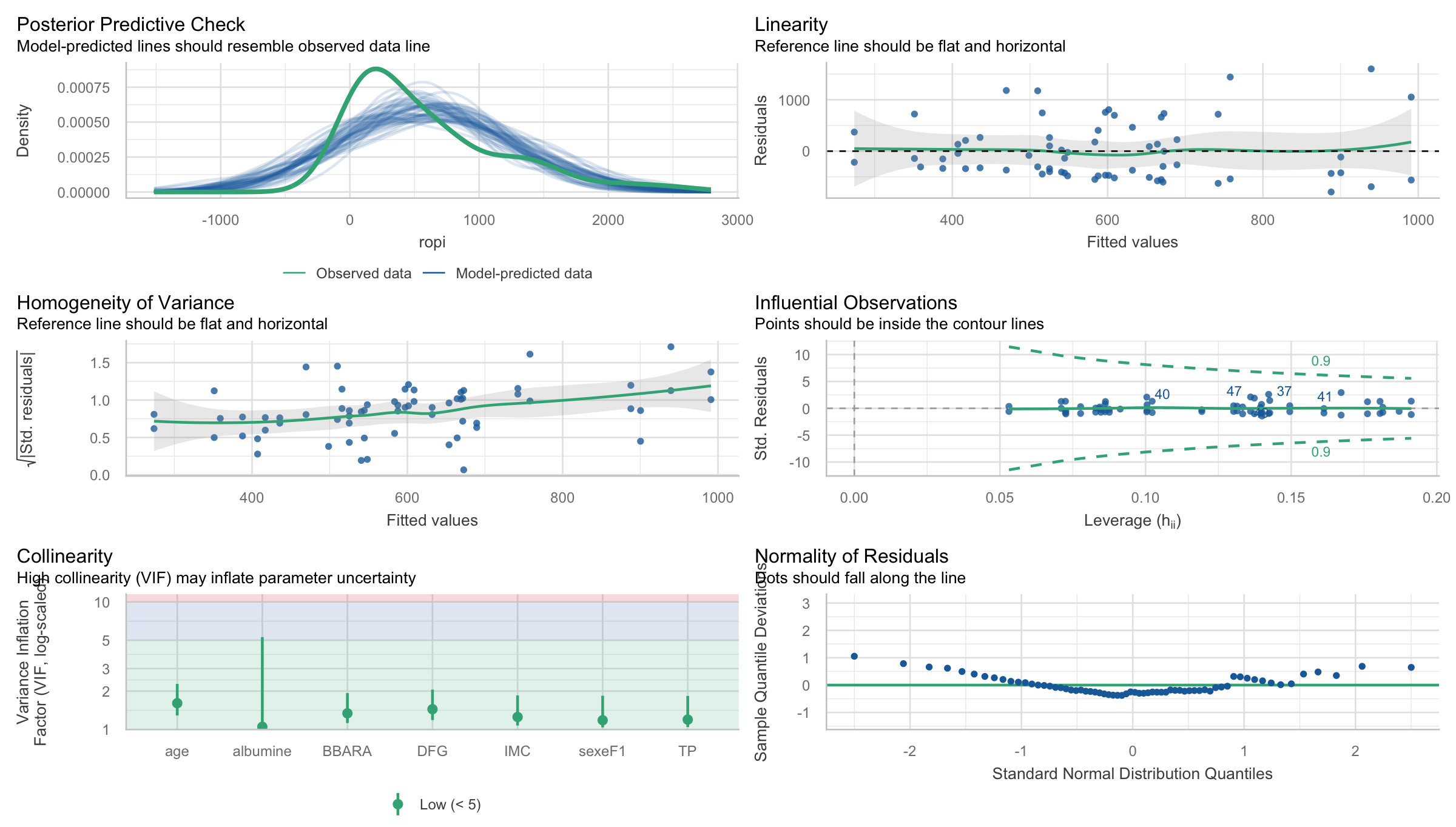
**
