# Supplementary material for: A prospective observational study of plasma concentrations and safety of combined intravenous lidocaine and epidural ropivacaine in laparotomy surgery
Source: PLoS One. 2026 Mar 6;21(3):e0344277. doi: 10.1371/journal.pone.0344277 (PMC12965542; doi:10.1371/journal.pone.0344277)
Supplement: S2 File — (DOCX) [file pone.0344277.s007.docx]

**Study protocol**

Evaluation of Plasma Concentrations of Intravenous Lidocaine and Epidural Ropivacaine When Used in Combination in Major Abdominal Surgeries

Acronym: LARA

Sponsor Code: PI2021_843_0197

EudraCT Number: 2021-005508-37 Category 1 Research Involving Human Subjects Protocol – Version 1.2 (dated 08/11/2021)

Favorable opinion from CPP (Ethics Committee) on 19/11/2021; Authorization from ANSM on 20/12/2021.

Protocol Update History

Version Date Reason for Update

1.0 25/02/2021 Submission to the CHU Amiens-Picardie Project Evaluation Committee

1.1 15/09/2021 Response to CEPR comments and submission to the CPP and ANSM.

1.2 08/11/2021 Response to CPP comments.

Sponsor:

CHU Amiens-Picardie – Clinical Research and Innovation Directorate

80054 Amiens Cedex 1, France

Tel: 03.22.08.83.70 – Fax: 03.22.08.96.45

Email: [collin.arnaud@chu-amiens.fr](mailto:collin.arnaud@chu-amiens.fr)

Methodology and Data Management Center:

Mr. Momar DIOUF – Biostatistician

Mr. Hassan BERRISSOUL – Data Manager

Mr. Salah MATTOUG – Project Manager

Clinical Research and Innovation Directorate, CHU Amiens-Picardie

80054 Amiens Cedex 1, France

Tel: 03.22.08.83.70 – Fax: 03.22.08.96.45

Coordinating Pharmacy:

Dr. Mohamed BELHOUT – Chief Pharmacist

Dr. Sophie BODDAERT – Head of Clinical Trials Sector (Hospital Pharmacist)

Dr. Florian SMAGGHE – Head of Clinical Trials Sector (Hospital Pharmacist)

Tel: 03.22.08.71.40 – Fax: 03.22.08.71.41

Email: [boddaert.sophie@chu-amiens.fr](mailto:boddaert.sophie@chu-amiens.fr)

Clinical Research Vigilance Unit:

Clinical Research and Innovation Directorate, CHU Amiens-Picardie

Tel: 03.22.08.83.90 – Fax: 03.22.08.96.45

Email: DRCI-vigilance@chu-amiens.fr

Principal Investigator:

Dr. Ottilie FUMERY-TROCHERIS – Anesthesiology and Intensive Care Department

Amiens University Hospital, 80054 Amiens Cedex, France

Tel: 03.22.08.78.36 – Fax: 03.22.45.53.40

Email: fumery.ottilie@chu-amiens.fr

Other Specialists (Co-Investigators):

Anne-Sophie HURTEL LEMAIRE, Sophie BODEAU, Dr. Youssef BENIS – Clinical Pharmacology Department, CHU Amiens-Picardie

Table of Contents

1. SUMMARY OF THE RESEARCH

2. ABSTRACT

3. SCIENTIFIC JUSTIFICATION AND GENERAL DESCRIPTION

3.1. Current State of Knowledge

3.1.1. On the pathology

3.1.2. On reference treatments/strategies/procedures and those under study

3.2. Research Hypotheses and Expected Results

3.3. Rationale for Methodological Choices

3.4. Benefit/Risk Assessment

3.5. Expected Outcomes

4. OBJECTIVES OF THE RESEARCH

4.1. Primary Objective

4.2. Secondary Objectives

5. ENDPOINTS

5.1. Primary Endpoint

5.2. Secondary Endpoints

6. STUDY DESIGN

6.1. Study Schematic

7. ELIGIBILITY CRITERIA

7.1. Inclusion Criteria

7.2. Exclusion Criteria

7.3. Feasibility and Recruitment Methods

8. STUDY TREATMENTS/STRATEGIES/PROCEDURES

8.1. Investigational Treatment/Strategy/Procedure

8.1.1. Study Drug Handling

8.1.1.1. Supply of Products

8.1.1.2. Product Packaging

8.1.1.3. Product Labeling

8.1.1.4. Dispensation of Products

8.1.1.5. Storage

8.1.1.6. Return and Destruction of Unused Products

8.1.2. Blinding (Masking)

8.1.2.1. Organization of Blinding

8.2. (Not Applicable – numbering adjusted to maintain structure)

9. ASSOCIATED TREATMENTS AND PROCEDURES

9.1. Allowed Associated Treatments/Procedures

9.2. Prohibited Associated Treatments/Procedures

10. CONDUCT OF THE STUDY

10.1. Study Schedule

10.2. Patient Follow-up Summary Table

10.3. Pre-Inclusion Visit

10.4. Randomization

10.5. Inclusion Visit

10.6. Follow-up Visits

10.7. End-of-Study Visit

10.8. Rules for Discontinuing the Study

10.8.1. Withdrawal of a Participant from the Study

10.8.2. Early Termination of Part or All of the Study

10.9. Study-Related Constraints and Participant Compensation

11. MANAGEMENT OF ADVERSE EVENTS AND NEW FINDINGS

11.1. Definitions

11.2. Description of Expected Serious Adverse Events

11.3. Procedures in Case of Adverse Event or New Finding

11.4. Reporting and Recording of Unexpected SAEs and New Findings

11.5. Annual Safety Report

12. STATISTICAL ASPECTS

12.1. Calculation of Study Size

12.2. Statistical Methods

13. STUDY MONITORING

14. ACCESS TO DATA AND SOURCE DOCUMENTS

14.1. Access to Data

14.2. Source Data

14.3. Data Confidentiality

15. QUALITY CONTROL AND ASSURANCE

15.1. Instructions for Data Collection

15.2. Study Monitoring (Site Visits)

15.3. Quality Control

15.4. Data Management

15.5. Audit and Inspection

16. ETHICAL AND REGULATORY CONSIDERATIONS

17. ARCHIVING OF STUDY DOCUMENTS AND DATA

18. PUBLICATION RULES

18.1. Scientific Communications

18.2. Communication of Results to Patients

18.3. Data Transfer

19. REFERENCES

List of Abbreviations

AL – Local Anesthetic(s)

ALR – Regional Anesthesia (Anesthésie Loco-Régionale)

APD – Epidural Anesthesia (Anesthésie Péridurale)

BIS – Bispectral Index

CPA – Pre-Anesthesia Consultation (Consultation Pré-Anesthésique)

ECG – Electrocardiogram

ETT – Transthoracic Echocardiography (Echographie Trans-Thoracique)

IV – Intravenous

IVSE – Intravenous via Infusion Pump (Intra-Veineux en Seringue Électrique)

TA – Arterial Blood Pressure (Tension Artérielle)

VPA – Pre-Anesthesia Visit (Visite Pré-Anesthésique)

1. SUMMARY OF THE RESEARCH

SPONSOR: CHU Amiens-Picardie PRINCIPAL INVESTIGATOR: Dr. Ottilie Fumery, Clinical Fellow in Anesthesiology

TITLE: Evaluation of plasma concentrations of intravenous lidocaine and epidural ropivacaine when used in combination in major abdominal surgeries.

ACRONYM: LARA

BACKGROUND/CONTEXT: Major abdominal surgeries cause intense pain in both the intraoperative and postoperative periods. Management of this pain relies on epidural analgesia on one hand and intravenous lidocaine on the other. These two techniques are often used together. However, currently no study has evaluated the safety of this combination in major abdominal surgeries.

OBJECTIVES: The primary objective of this study is to evaluate the plasma concentrations of lidocaine and ropivacaine during the combination of an epidural anesthesia and intravenous lidocaine in a major abdominal surgery. The secondary objective is to investigate the undesirable side effects related to local anesthetics when combining epidural anesthesia with intravenous lidocaine during a major abdominal surgery (central nervous system effects: seizures, dizziness, convulsions, paresthesia, tongue numbness, hyperacusis, tinnitus, visual disturbances, dysarthria, muscle contractions, tremors; hypoesthesia; cardiac disorders: bradycardia, tachycardia, conduction disturbances possibly progressing to cardiac arrest). STUDY DESIGN: Before general anesthesia, patients undergoing major abdominal surgery via laparotomy will have a thoracic epidural catheter placed; however, no injection through the catheter will occur during the surgery. During surgery, patients will receive an intravenous bolus of lidocaine at 1.5 mg/kg (SFAR recommendations), followed by a continuous IV infusion of lidocaine at 2 mg/kg/h (SFAR recommendations), which will be stopped 30 minutes before the end of surgery (at the time of surgical closure). The epidural ropivacaine infusion will be started at the same time the lidocaine infusion is stopped, using ropivacaine 1 mg/mL with an initial minimum bolus of 5 mL, then maintained at a minimum rate of 5 mg/h via the epidural catheter for up to 24 hours postoperatively. The rest of the anesthetic protocol is at the discretion of the anesthesiologist in charge of the patient. As part of the study, blood samples will be taken to measure lidocaine plasma levels: 30 minutes after the induction bolus, at the time the continuous lidocaine infusion is stopped, and 2 hours after the start of the epidural ropivacaine infusion. Blood samples to measure ropivacaine plasma levels will also be taken 2 hours after the start of the epidural and 24 hours after the start of the epidural.

INCLUSION CRITERIA:

Patient undergoing major abdominal surgery with laparotomy

Age ≥ 18 years

Written informed consent obtained

Affiliated to a social security system (health insurance)

EXCLUSION CRITERIA:

Major abdominal surgery via laparoscopic approach

Contraindication to thoracic epidural placement (e.g. coagulation disorders, localized or systemic infection, shock or uncorrected hypovolemia, respiratory failure if block level > T7, severe aortic or mitral stenosis, obstructive cardiomyopathy, severe uncompensated heart failure)

Allergy to local anesthetics; Contraindication to IV lidocaine (acute porphyria, unpaced atrioventricular conduction disorders, severe heart failure, severe liver failure, uncontrolled epilepsy, treatment with Class I antiarrhythmics)

Contraindication to ropivacaine (allergy to local anesthetics, acute porphyria)

Major hepatectomy with vascular clamping

Patient refusal to participate

Minor (age < 18)

Patient under guardianship/curatorship or deprived of liberty

Pregnant, parturient, or breastfeeding women

Cognitive impairment precluding giving informed consent

STUDY TREATMENT/PROCEDURE: Induction with IV lidocaine 1.5 mg/kg followed by continuous infusion at 2 mg/kg/h (per SFAR guidelines), stopped at surgical closure. Immediately at discontinuation, initiation of thoracic epidural analgesia with ropivacaine, infused for 24h at a minimum of 5 mg/h after an initial minimum bolus of 5 mg via the epidural catheter. As part of the study, blood samples will be taken for lidocaine levels 30 minutes after the induction bolus, at the end of the continuous infusion, and 2 hours after starting the epidural ropivacaine infusion. Blood samples for ropivacaine levels will be taken 2 hours after starting the epidural and 24 hours after starting the epidural.

ENDPOINTS: The primary endpoint is the plasma concentration of ropivacaine and lidocaine falling outside the safe range at the various time points of management. For lidocaine, the safe plasma range is 1.4 to 6 µg/mL; neurotoxicity appears from 15 µg/mL and cardiotoxicity from 21 µg/mL. For ropivacaine, the usual maximum safe venous concentration is around 2.2 µg/mL. The secondary endpoint is the occurrence of clinical systemic toxicity of local anesthetics within 48 hours postoperatively, defined by any of the following events: central nervous system disturbances (seizures, dizziness, paresthesia, tongue numbness, hyperacusis, tinnitus, visual disturbances, dysarthria, muscle twitching, tremors, hypoesthesia) or cardiac disturbances (bradycardia, tachycardia, conduction disorder potentially progressing to cardiac arrest). This composite endpoint will be counted if at least one of the above events is present.

SAMPLE SIZE: 50 patients

NUMBER OF CENTERS: Single-center (CHU Amiens-Picardie)

STUDY DURATION:

Inclusion period: 12 months

Participation duration for each patient: 48 hours

Total study duration: 12 months

STATISTICAL ANALYSIS: We expect a low percentage of patients with ropivacaine or lidocaine levels outside the safe range. Assuming this percentage is about 10%, enrolling 50 patients would allow us to estimate this percentage with a confidence interval no wider than 16% (±8% precision). In any case, the percentage can be estimated with a precision no worse than ±14% (the worst precision corresponds to an incidence around 50%). EXPECTED

OUTCOMES: Demonstrating the safety of the lidocaine + epidural ropivacaine combination in laparotomy will support standardizing this practice. It will affirm and provide evidence that combining intravenous lidocaine with epidural analgesia using ropivacaine in major open abdominal surgery is safe, thereby encouraging uniform adoption of this analgesic strategy.

2. ABSTRACT

Title: Evaluation of plasma concentrations of intravenous lidocaine and epidural ropivacaine when used in combination in major abdominal surgery. CHU Amiens-Picardie is the sponsor of this research. This research will be conducted with the support of [name of pharmaceutical company] / [source of funding (e.g., PHRC, internal grant, etc.)].

Background: Major abdominal surgery by laparotomy often requires effective analgesia. Thoracic epidural analgesia (TEA) is the gold standard for postoperative pain management in this setting and can be combined with intravenous lidocaine or used as an alternative when TEA is contraindicated, in order to reduce perioperative opioid use (which can cause side effects such as ileus or nausea/vomiting and delay enhanced recovery). Intravenous lidocaine and TEA share several properties (anti-hyperalgesic, anti-inflammatory, pro-kinetic for the intestines, anti-tumoral effects, etc.), suggesting an additive effect of their combination, which has not yet been studied.

Purpose: To demonstrate that thoracic epidural analgesia combined with intravenous lidocaine is safe and does not lead to adverse events during major abdominal surgeries.

Primary Outcome: Repeated measurements of plasma lidocaine and ropivacaine levels in the perioperative period.

Secondary Outcomes: Occurrence of adverse events attributable to local anesthetics – signs of neurotoxicity (e.g., tremors, seizures) and/or cardiotoxicity (e.g., conduction disturbances, arrhythmias, or the rare event of cardiopulmonary arrest).

Study Design: Lidocaine will be administered as an IV bolus during anesthetic induction and infused continuously until surgical closure (stopped ~30 minutes before the end). At the time of stopping lidocaine, ropivacaine will be injected through the thoracic epidural catheter and then infused for 24h. The occurrence of adverse events will be monitored through clinical examination and by measuring plasma lidocaine and ropivacaine levels during the perioperative period.

Eligibility Criteria:

Inclusion: Major abdominal surgery via laparotomy.

Exclusion: Laparoscopic approach, any contraindication to TEA, contraindication to ropivacaine or IV lidocaine.

Interventions: Blood samples for lidocaine level at 30 min before end of surgery, at end of infusion, and 2h after start of epidural ropivacaine; blood samples for lidocaine and ropivacaine levels 2h after start of epidural; and blood sample for ropivacaine level 24h after start of epidural infusion.

Number of subjects: 50 patients.

3. SCIENTIFIC JUSTIFICATION AND GENERAL DESCRIPTION

3.1. Current State of Knowledge

3.1.1. On the Pathology

Major abdominal surgeries can be performed via laparotomy when a minimally invasive (coelioscopic) approach is not possible. Laparotomy involves a large incision through the abdominal skin and musculature to access the internal organs, and it causes significant pain that delays the patient’s postoperative recovery. Managing pain in these patients is therefore a major concern for perioperative care providers. Pain control relies on two main techniques: epidural anesthesia and intravenous lidocaine.

3.1.2. On Reference Treatments/Strategies/Procedures and Those Under Study

Epidural anesthesia (EPA) is the gold standard for managing laparotomy pain. However, it induces sympathetic blockade and can cause hypotension, which may be dangerous for the patient and lead to intra- or postoperative organ dysfunction. Therefore, the infusion of local anesthetics through the epidural catheter is typically started only after the high-risk surgical stages are over – in practice, at the time of surgical closure. Intravenous lidocaine has multiple effects: anti-arrhythmic, anesthetic, analgesic, anti-inflammatory, antithrombotic, bronchodilatory, neuroprotective, and even antimicrobial properties. IV lidocaine infusion does not cause vasoplegia, whereas epidural anesthesia blocks sympathetic nerves and can reduce vascular resistance. The clinical analgesic efficacy of IV lidocaine has been confirmed by a 2018 meta-analysis. It is administered at the start of surgery (during induction) and continued intraoperatively until closure, at which point it is stopped. IV lidocaine via infusion pump is thus proposed as an alternative to epidural analgesia when the latter is contraindicated or if epidural placement fails. Depending on the chosen analgesic technique, there is a period not covered by local anesthetics: for lidocaine, this gap is at closure and immediate postoperative; for epidural, it is almost the entire intraoperative period until closure. This is why practitioners at our university hospital combine these two techniques. We know anecdotally from discussions with colleagues that this combined approach is also used in other hospitals, but no study has been conducted on the subject. However, there is a theoretical risk of local anesthetic toxicity with this combination, which dissuades some clinicians from using both techniques despite the potential significant benefit for patients. The combination of two local anesthetics is feasible only if we consider the kinetics of their plasma concentrations with each technique. Lidocaine is administered IV, whereas ropivacaine is administered into the epidural space. The systemic absorption of ropivacaine infused epidurally has been studied in several works, which showed plasma concentrations very far from toxic thresholds. This finding is reassuring for its concomitant use with IV lidocaine. However, we must consider whether there is any pharmacodynamic potentiation between these two drugs. Before attempting to demonstrate any clinical advantage of this combination for patient care, it is imperative to first ensure that combining them does not increase their toxicity. To date, although this combination is in widespread use, no study has been carried out to demonstrate its safety. Our study aims to show that toxic plasma thresholds are not reached when these two techniques are combined. We will also monitor for clinical signs of local anesthetic toxicity (central nervous system effects: seizures, dizziness, paresthesia, tongue numbness, hyperacusis, tinnitus, visual disturbances, dysarthria, muscle contractions, tremors; hypoesthesia; cardiac effects: bradycardia, tachycardia, conduction disturbance potentially progressing to cardiac arrest) when they are used together.

3.2. Research Hypotheses and Expected Results

Our hypothesis is that combining continuous IV lidocaine infusion with epidural anesthesia during management of a major abdominal surgery does not increase the plasma concentrations of local anesthetics beyond toxic thresholds. Through repeated blood sampling, we hope to show that plasma levels of the local anesthetics remain well below toxic values. Furthermore, by observing an absence of adverse events related to local anesthetic use (central nervous system effects – which are rare – such as seizures, dizziness, loss of consciousness, paresthesia, tongue numbness, hyperacusis, tinnitus, visual disturbances, dysarthria, muscle tremors; hypoesthesia; and cardiac effects such as bradycardia, tachycardia, conduction disturbances up to cardiac arrest), we hope to demonstrate no cumulative cardiac or neurological toxicity when both are used together.

3.3. Rationale for Methodological Choices

Lidocaine and ropivacaine are both metabolized in the liver. The toxic thresholds for these two local anesthetics are becoming well known: for lidocaine, the therapeutic plasma range is 1.4 to 5 µg/mL, and toxicity can begin at concentrations above 5 µg/mL. Neurotoxic effects appear around 15 µg/mL and cardiotoxicity around 21 µg/mL. For ropivacaine, the usual maximal total venous concentration not to exceed is about 2.2 µg/mL. The half-life of IV lidocaine is 80–110 minutes. Ropivacaine, when administered into the epidural space, is absorbed completely in a biphasic manner, with half-lives of approximately 14 minutes and 4 hours for the two phases in adults. Its slow absorption is the rate-limiting step in its elimination, leading to a longer apparent elimination half-life after epidural administration compared to IV administration. In our study, five plasma measurements of lidocaine and ropivacaine will be taken at different times during surgery and up to 24 hours post-op: lidocaine level at 30 minutes after the bolus, lidocaine level at the end of its infusion (i.e. at surgical closure), lidocaine level at 2 hours after starting the epidural, ropivacaine level at 2 hours after starting the epidural, and ropivacaine level at 24 hours after starting the epidural. Achieving a steady plasma level during a continuous infusion takes more than 60 minutes, which is why an initial bolus is recommended. Given the rapid distribution after a bolus, it’s recommended to start the maintenance continuous infusion promptly if a stable plasma concentration is to be maintained for infusions longer than 20 minutes. This justifies our first blood draw at 30 minutes after the lidocaine bolus. Thereafter, the plasma concentration remains stable around a level determined by the dose and infusion rate, which is why we will measure at the moment of stopping the infusion to capture the intraoperative plasma level of lidocaine. For the epidural, systemic absorption is gradual. Studies show a linear increase in plasma levels during the continuous infusion; the 2-hour ropivacaine level will provide an initial indication of ropivacaineemia. The lidocaine sample taken at the same 2-hour mark will indicate the residual lidocaine concentration at the time ropivacaine levels are rising, giving insight into the overlap period of both local anesthetics, and whether lidocaine’s decline follows the expected pattern when another local anesthetic is present. Finally, the ropivacaine level at 24 hours post-epidural induction will reflect the average plasma concentration after a full day of continuous epidural ropivacaine infusion. Lidocaine is not measured at 24h because it will have been fully eliminated by that time.

3.4. Benefit/Risk Assessment

Demonstrating the safety of this combination will firstly allow us to standardize our practices. It will then enable further research to demonstrate the superiority of combining these two techniques compared to epidural analgesia alone or IV lidocaine alone. In this way, we could improve recovery after these major surgeries that cause severe pain and morbidity. The local anesthetics will be used according to current recommendations and good practice guidelines, and their usage will not be altered by this research. The only additional risk induced by this study is related to the extra blood samples beyond the routine tests normally performed during patient care. Blood draws will be done through the arterial catheter (placed as usual for the surgery). This arterial line is in most cases kept for 24h (when patients are in a step-down or intensive care unit). If the arterial line is removed earlier, the 24h post-epidural ropivacaine sample will be taken by a nurse via venipuncture at the investigator’s request (or by the investigator).

3.5. Expected Outcomes

Demonstrating the safety of this combination will initially allow us to standardize our practices. Combining IV lidocaine analgesia with epidural anesthesia appears very promising for managing patients undergoing major abdominal surgery, as this combination can modulate pain via different pathways. Currently, no study has shown the superiority of this combination over other analgesic techniques. Proving that no additional adverse effects occur when these two techniques are combined will allow those comparative studies to be performed and help identify the best therapeutic strategy for patients. This could optimize patients’ postoperative recovery.

4. OBJECTIVES OF THE RESEARCH

4.1. Primary Objective

The primary objective of this study is to evaluate the plasma concentrations of lidocaine and ropivacaine when an epidural anesthesia is combined with intravenous lidocaine during a major abdominal surgery.

4.2. Secondary Objectives

The secondary objective is to identify any undesirable side effects related to local anesthetics in connection with the combination of epidural anesthesia and intravenous lidocaine during a major abdominal surgery. Specifically, we will look for signs of local anesthetic toxicity, including central nervous system symptoms (seizures, dizziness, loss of consciousness/“attacks,” paresthesia, tongue numbness, hyperacusis, tinnitus, visual disturbances, dysarthria, muscle contractions, tremors, hypoesthesia) and cardiac symptoms (bradycardia, tachycardia, conduction disturbances up to cardiac arrest) potentially attributable to this combination.

5. ENDPOINTS

5.1. Primary Endpoint

The primary endpoint is the plasma concentration of ropivacaine and lidocaine falling outside the safe range at the various time points of patient management. We will obtain five plasma measurements of lidocaine and ropivacaine at different times during surgery and up to 24 hours postoperatively: lidocaine level at 30 minutes after the bolus injection, lidocaine level at the end of its infusion (i.e. at surgical closure), lidocaine level at 2 hours after induction of the epidural, ropivacaine level at 2 hours after induction of the epidural, and ropivacaine level at 24 hours after induction of the epidural. The toxic thresholds of these two local anesthetics are known: for lidocaine, the safe plasma range is 1.4 to 6 µg/mL; neurotoxicity appears from 15 µg/mL and cardiotoxicity from 21 µg/mL. For ropivacaine, the usual maximum total venous concentration not to exceed is around 2.2 µg/mL. Any single measurement above the toxicity threshold will be considered an occurrence of toxicity.

5.2. Secondary Endpoints

The secondary endpoint is the occurrence (yes/no) of adverse events related to the use of local anesthetics, defined as any of the following:

Central nervous system symptoms: seizures, dizziness, loss of consciousness (“attacks”), paresthesia, numbness of the tongue, hyperacusis, tinnitus, visual disturbances, dysarthria, muscle contractions, tremors. Cardiac symptoms: bradycardia, tachycardia, conduction disorder possibly progressing to cardiac arrest. (If at least one of the above events occurs, it will count toward the endpoint.)

6. STUDY DESIGN

6.1. Study Schematic

This is a prospective, descriptive, single-center study. Patients undergoing major abdominal surgery via open laparotomy are included. The epidural catheter is placed at the beginning of the procedure, before induction of general anesthesia. An IV lidocaine bolus of 1.5 mg/kg is given at induction, then continued via infusion pump at 2 mg/kg/h. If the patient is obese (BMI > 30), the dosage will be adjusted based on ideal body weight as a precaution, although there are no specific guidelines on this point. The lidocaine infusion is stopped approximately 30 minutes before the end of surgery. At that same time, the epidural is “activated,” meaning an initial bolus of at least 5 mL of ropivacaine 1 mg/mL is given through the epidural, followed by a continuous infusion starting at a minimum rate of 5 mg/h. This rate will then be adjusted according to the patient’s pain in the recovery room. The total amount of ropivacaine administered will be recorded. Blood samples will be taken for lidocaine level 30 minutes after the bolus, at the end of the infusion, and 2 hours after the start of the epidural ropivacaine infusion. Blood samples for ropivacaine level will be taken 2 hours after the start of the epidural and 24 hours after the start of the epidural. Blood draws will be done through the arterial catheter placed for surgery. In the majority of cases, this catheter is maintained for 24h (patients are admitted postoperatively to a continuous care or intensive care unit). If the arterial line is removed earlier, the 24h ropivacaine sample will be obtained by venous puncture by the ward nurse or investigator. Each blood draw requires 6 mL of blood. The remainder of the anesthetic and analgesic protocol (besides the lidocaine and epidural as described) is left to the discretion of the anesthesiologist in charge of the patient. Patients will be followed for 48 hours postoperatively. Any adverse events related to local anesthetics will be monitored (clinically and with appropriate monitoring such as continuous ECG, etc.).

7. ELIGIBILITY CRITERIA

7.1. Inclusion Criteria

Any patient scheduled for major abdominal surgery via laparotomy.

Age ≥ 18 years.

Patient has given informed consent in writing.

Patient is affiliated with a social security system (health insurance).

7.2. Exclusion Criteria

Exclusion criteria are:

Abdominal surgery via laparoscopy.

Contraindication to high lumbar/thoracic epidural placement (e.g., coagulation disorder, localized or systemic sepsis, shock or uncorrected hypovolemia, respiratory failure if block level above T7, severe aortic or mitral stenosis, obstructive cardiomyopathy, severe uncompensated heart failure, infection at puncture site).

Contraindication to IV lidocaine: allergy to local anesthetics, acute porphyria, unpaced atrioventricular conduction disorders, severe heart failure, severe liver failure, uncontrolled epilepsy, concomitant therapy with class I antiarrhythmic.

Contraindication to ropivacaine: allergy to local anesthetics, acute porphyria.

Failure to successfully place the epidural.

Major hepatectomy with vascular clamping.

Patient under 18 years of age.

Patient under legal protection (guardianship) or deprived of liberty.

Patient who is pregnant or breastfeeding.

Patient refusal to participate.

Patient not affiliated with a social security system.

7.3. Feasibility and Recruitment Methods

The possibility of participating in the protocol will be discussed during the Pre-Anesthesia Consultation (CPA), which is scheduled between 2 months and 48 hours before the planned surgery (on average about 3 weeks before surgery at CHU Amiens-Picardie). During this consultation, the protocol procedures will be explained to the patient. The informed consent will be obtained at the Pre-Anesthesia Visit (typically the day before surgery), to allow the patient time to consider their participation. Based on our current surgical caseload, we estimate that we can recruit the planned number of patients (50) within 12 months.

8. STUDY TREATMENTS/STRATEGIES/PROCEDURES

8.1. Investigational Treatment/Strategy/Procedure

Upon the patient’s arrival in the operating room, a thoracic epidural is placed by the anesthesiologist. This involves inserting the catheter into the epidural space, but initially no local anesthetic is injected through the epidural catheter. The conduct of general anesthesia—its induction and maintenance—is left to the discretion of the attending anesthesiologist in the operating room. At induction, lidocaine is administered intravenously at 1.5 mg/kg as a bolus, followed by a continuous IV infusion at 2 mg/kg/h until surgical closure, in accordance with SFAR guidelines. The use of intravenous lidocaine in this manner is consistent with its approved labeling. At surgical closure, the lidocaine infusion is stopped, and the epidural is activated with ropivacaine 1 mg/mL, given as an initial bolus of at least 5 mL and then continued at a minimum rate of 5 mg/h for 24 hours postoperatively. The use of ropivacaine in this manner is also in line with its approved labeling. As part of this study, blood samples will be taken to measure plasma lidocaine levels at: 30 minutes after the induction bolus, at the end of the continuous infusion, and 2 hours after the start of the epidural ropivacaine infusion. Blood samples to measure plasma ropivacaine levels will also be taken: 2 hours after the start of the epidural, and 24 hours after the start of the epidural. Each blood sample is 6 mL.

8.1.1. Study Drug Handling

8.1.1.1. Supply of Products

All medications used in the study are approved (have marketing authorization in France). Ropivacaine and lidocaine will be supplied by the hospital pharmacy of CHU Amiens-Picardie.

8.1.1.2. Product Packaging

The packaging will be the standard commercial packaging (as authorized by their marketing approvals) and will not be modified for the study.

8.1.1.3. Product Labeling

The labeling of the products will not be modified.

8.1.1.4. Dispensation of Products

Ropivacaine and lidocaine are routinely used and stored in the department by trained healthcare staff. They will be used in accordance with their approved indications. Staff will document their use in the patient’s medical record, noting the dose administered and the batch number used.

8.1.1.5. Storage

The drug units are stored in the clinical department under the conditions described in their Summary of Product Characteristics (SPC).

8.1.1.6. Return and Destruction of Unused Products

Used treatment units (e.g., empty vials or bags) are destroyed immediately after use by the clinical department via the usual biohazard waste disposal route. Any unused study drug (if applicable) will be disposed of similarly. There is no provision for returning unused study drugs to the pharmacy; they will be discarded per hospital protocol.

8.1.2. Blinding (Masking)

8.1.2.1. Organization of Blinding

Not applicable – this study is not blinded (open-label).

8.2. Associated Treatments and Procedures (if any)

Not applicable to this study

(Section numbering is maintained for structural consistency.)

9. ASSOCIATED TREATMENTS AND PROCEDURES

9.1. Allowed Associated Treatments/Procedures

Each patient will undergo general anesthesia conducted as per the usual care for this type of surgery. The choice of anesthetic protocol and any adjunct analgesic medications is left to the discretion of the anesthesiologist responsible for the patient on the day of surgery.

9.2. Prohibited Associated Treatments/Procedures

There are no prohibited procedures in this study; no specific co-interventions are disallowed beyond standard contraindications.

10. CONDUCT OF THE STUDY

10.1. Study Schedule

Start of inclusions: Immediately after obtaining the required regulatory approvals.

Inclusion period duration: 12 months.

Duration of participation for each patient: 48 hours.

Total study duration: 12 months.

10.2. Patient Follow-up Summary Table

The following table summarizes the timing of study-related procedures for each patient:

| Procedure / Assessment | Pre-inclusion (Day -60 to -2) | Inclusion (Day 0) | Visit 1 (30 min after lidocaine bolus) | Visit 2 (At surgical closure) | Visit 3 (2 h post-surgery) | Visit 4 (24 h post-surgery) | Visit 5 (Post-op Day 2) |
| --- | --- | --- | --- | --- | --- | --- | --- |
| Patient information | ✓ | ✓ |  |  |  |  |  |
| Informed consent |  | ✓ |  |  |  |  |  |
| Lidocaine level (lidocainemia) |  |  | ✓ | ✓ | ✓ |  |  |
| Ropivacaine level (ropivacainemia) |  |  |  |  | ✓ | ✓ |  |
| Adverse event monitoring |  |  | ✓ | ✓ | ✓ | ✓ | ✓ |

10.3. Pre-Inclusion Visit

The pre-inclusion visit is carried out by the investigator (study physician). It takes place between 2 months and up to 2 days before the inclusion visit, during the Pre-Anesthesia Consultation (CPA). All patients who are potential candidates (meeting inclusion criteria and no exclusion criteria) will be offered participation in the study. Information: During the pre-inclusion visit, the investigator informs the patient about the study and answers all questions regarding the study’s objective, the nature of the constraints, foreseeable risks, and expected benefits. The investigator also explains the patient’s rights in the context of research and verifies eligibility criteria. A copy of the information sheet and consent form is given to the patient at this time.

10.4. Randomization

There is no randomization in this study (it is a single-arm descriptive study).

10.5. Inclusion Visit

This takes place no later than the day before the surgery, conducted by the investigator. Consent acquisition: During the pre-inclusion visit (CPA), the investigator provided all the study information to the patient and answered questions about the study’s objectives, constraints, risks, and potential benefits, as well as explained the patient’s rights in a biomedical research context. The eligibility criteria were also verified. The patient was given the information sheet and consent form to review. After this information session, the patient is given time to consider their decision. The investigator is responsible for obtaining the patient’s written informed consent. The consent form must be signed before any study-specific clinical or paraclinical examination is performed.If the patient agrees to participate, both the patient and the investigator will print their names, date, and sign the consent form. The distribution of the information sheet and consent copies is as follows:

One signed copy of the information sheet and consent is given to the patient.

The original signed consent form is kept by the investigator (even if the patient moves or transfers care during the study) in a secure location inaccessible to third parties.

The patient’s medical history, clinical examination, and any usual care paraclinical exams (e.g., cardiology consult, TTE, ECG, liver function tests, gastroenterology consult, etc.) will be reviewed to ensure no contraindications to epidural or the use of the study anesthetics are present, both at the CPA and confirmed at the Pre-Anesthesia Visit (usually the day before surgery). Specifically, for the epidural: no coagulation disorders, no localized or systemic infection, no shock or uncorrected hypovolemia, no respiratory failure if high block, no severe aortic/mitral stenosis, no obstructive cardiomyopathy, no severe uncompensated heart failure. For lidocaine: ensure no allergy to local anesthetics, no acute porphyria, no unpaced AV block, no severe heart failure, no severe liver failure, no uncontrolled epilepsy. For ropivacaine: ensure no allergy to local anesthetics or acute porphyria.

10.6. Follow-up Visits

Follow-up begins once anesthesia induction is done and the lidocaine bolus has been administered. Clinically, this involves continuously monitoring and checking for any occurrence of local anesthetic adverse effects (cardiac and/or neurotoxicity) via physical exam and patient monitors (continuous ECG, BIS, invasive or non-invasive blood pressure, etc.) throughout the surgery and up to 24 hours postoperatively, whether the patient is in a standard ward or in ICU. We will also perform plasma measurements of lidocaine and ropivacaine at specific perioperative times: three lidocaine levels in total – the first 30 minutes after the induction bolus, one at the time the infusion is stopped (surgical closure), and the last one 2 hours after the epidural ropivacaine bolus; and two ropivacaine levels – the first 2 hours after the epidural bolus (at the same time as the last lidocaine level) and the second at 24 hours after epidural initiation.

10.7. End-of-Study Visit

The end-of-study visit corresponds to the last follow-up visit (Visit 5), which is performed on postoperative Day 2.

10.8. Rules for Discontinuing the Study

10.8.1. Withdrawal of a Participant from the Study

Any participant is free to withdraw from the study at any time, for any reason, upon request, without prejudice to their subsequent care. In the event of withdrawal, the ongoing standard treatment will be continued exactly as for patients not in the study, as participation in this study does not alter the therapeutic management. The date of withdrawal and the reason (if provided) will be recorded in the case report form (CRF) by the investigator. Every effort will be made to adhere to the protocol’s procedures. However, the clinician in charge of the patient may deviate from the protocol at any time if deemed necessary, particularly in the event of serious adverse events. Any such decision and its justification must be documented in the CRF. In case of major protocol deviations (regarding regulatory aspects, eligibility criteria, primary endpoint collection, etc.), the participant’s data may be excluded from analysis.

10.8.2. Early Termination of Part or All of the Study

The study may be terminated prematurely in the event of unexpected serious adverse events (with or without necessitating a review of the product’s safety profile), or if unforeseen events or new information about the product suggest that the study objectives are unlikely to be achieved. Such circumstances may lead the sponsor to halt the study early. CHU Amiens-Picardie (the sponsor) reserves the right to stop the study at any time if it becomes apparent that enrollment objectives are not being met. In case of premature discontinuation of the study, the sponsor will inform the ANSM and the Ethics Committee (CPP) within 15 days.

10.9. Study-Related Constraints and Participant Compensation

A person participating in this research may take part in another study concurrently, except they may not participate in another interventional therapeutic trial, for a period of 2 days after the surgery. No compensation is planned for participants in this study.

11. MANAGEMENT OF ADVERSE EVENTS AND NEW FINDINGS

11.1. Definitions

Adverse Event (AE) (per Article R1123-46 of the Public Health Code): Any harmful event occurring in a person participating in a research study involving human subjects, whether or not the event is related to the research or the product under study

.

Adverse Effect (per Article R1123-46): An adverse event occurring in a person participating in a research study, when that event is related to the research or the product under study.

Serious Adverse Event (SAE): Any adverse event that:

• results in death,

• is life-threatening,

• requires hospitalization or prolongation of existing hospitalization,

• causes a significant or persistent disability or incapacity,

• causes a congenital anomaly or birth defect,

• or any event considered medically serious (for a drug, regardless of dose administered). (“Life-threatening” refers to an immediate risk of death at the time of the event.)

Unexpected Adverse Effect:

For drug studies: any adverse effect of the drug that is not consistent in nature, severity, frequency, or outcome with the reference safety information (as in the Summary of Product Characteristics or the Investigator’s Brochure for an unauthorized product).

For studies on a medical device or in vitro diagnostic device: any adverse effect of the device not consistent in nature, severity, or outcome with the information in the device’s instructions for use or Investigator’s Brochure.

For other human research (not involving drugs or devices): any adverse effect whose nature, severity, or outcome is not consistent with the information relating to the products, procedures, or methods used in the research.

New Finding (Fait nouveau) (Article R1123-46): Any new information that could lead to a reassessment of the benefit/risk ratio of the research or the product under study, or that could warrant changes in the use of the product, in how the research is conducted, or in the documents related to the research, or could justify suspending, interrupting, or modifying the research protocol.

11.2. Description of Expected Serious Adverse Events

The expected serious adverse events in this context are those related to the practice of regional anesthesia and the use of IV lidocaine and epidural ropivacaine. These include systemic toxicity from local anesthetics, severe allergic reactions to local anesthetics, and infections at the epidural puncture site.

11.3. Procedures in Case of Adverse Event or New Finding

The investigator will assess each adverse event for its severity. The investigator must notify the sponsor without delay upon becoming aware of any serious adverse event or any new finding, if it occurs:

from the date of consent signature, during the entire planned follow-up duration for the patient in the study, indefinitely, if the event might be attributable to the investigational treatment.

Notification should follow these guidelines:

| Type of Event | Notification Method | Timeline for Notification to Sponsor |
| --- | --- | --- |
| Non-serious AE | Document in the CRF | No immediate notification (report in CRF only) |
| SAE (expected) | Initial SAE report form (+ written report if needed) | Immediate notification to sponsor |
| SAE (unexpected) | Initial SAE report form (+ written report if needed) | Immediate notification to sponsor |
| New Finding | New finding report form (+ written report if needed) | Immediate notification to sponsor |
| Pregnancy (during or shortly after study) | Pregnancy notification form | Upon confirmation of pregnancy |

Sponsor / Vigilance Unit Contact:

Clinical Research and Innovation Directorate, CHU Amiens-Picardie

Phone: 03 22 08 83 90 – Fax: 03 22 08 96 45

Email: DRCI-vigilance@chu-amiens.fr All such events will be followed until complete resolution. Additional information (via follow-up report form) regarding the outcome of the event, if not known at the time of the initial report, will be sent to the sponsor by the investigator. Note: Occurrence of a pregnancy during or immediately after the study is not in itself classified as an SAE. However, any pregnancy must be reported in the same manner as an SAE because it requires special follow-up. Any abnormality observed in the fetus or child will be reported. Any voluntary termination of pregnancy (elective abortion), medically indicated termination, or miscarriage must be reported via a pregnancy form, and if it required hospitalization, it should also be reported as an SAE following the same timelines.

11.4. Reporting and Recording of Unexpected SAEs and New Findings

The sponsor (or designated vigilance unit) will report all unexpected SAEs and new findings that occur during the study to the ANSM and the competent Ethics Committee (CPP) within the following timeframes:

For an unexpected serious adverse effect that results in death or is life-threatening: without delay upon the sponsor becoming aware of it.

For other unexpected serious adverse effects: no later than 15 days from when the sponsor became aware of them. The sponsor will submit relevant follow-up information in the form of a safety report to ANSM. In the case of a suspected unexpected serious adverse reaction (SUSAR) that resulted in death or was life-threatening, follow-up information will be reported within 8 days of the initial report; for other SUSARs or a new finding, follow-up information will be reported within 8 days after the 15-day deadline mentioned above. The Ethics Committee (CPP) will ensure, if necessary, that participants are informed of adverse events and that they reconfirm their consent to continue in the study. For drug studies, the sponsor/vigilance unit will also record all unexpected SAEs in the EudraVigilance database. In the case of a blinded study (not applicable here), the sponsor would break the blind for reporting SAEs to ANSM and CPP

11.5. Annual Safety Report

On the anniversary date of the study’s regulatory authorization, the sponsor will prepare an annual safety report that includes:

A list of all serious adverse events that are deemed to be related to the investigational drug(s) (including both expected and unexpected serious events).

A concise, critical analysis of the safety of participants enrolled in the research.

This report will be sent to ANSM and the CPP within 60 days after the anniversary date of the study’s authorization

12. STATISTICAL ASPECTS

12.1. Calculation of Study Size

We anticipate that the proportion of patients with ropivacaine or lidocaine concentrations outside the safe range will be low. Assuming this proportion is around 10%, enrolling 50 patients would allow us to estimate this percentage with a confidence interval whose width does not exceed 16% (i.e., ±8% precision).

In any scenario, the percentage can be estimated with a precision no worse than ±14% (the worst-case precision corresponds to a proportion near 50%).

12.2. Statistical Methods

Quantitative variables will be described as mean ± standard deviation and median [range]. Qualitative variables will be described as percentages with 95% confidence intervals

Primary endpoint analysis: We will calculate the percentage of patients who have at least one plasma concentration of ropivacaine or lidocaine outside the safe range. A 95% confidence interval (using an asymptotic or exact method as appropriate) will be computed for this percentage.

Secondary endpoint analysis: The percentage of patients experiencing a toxic event will be calculated with a 95% confidence interval (asymptotic or exact method). All statistical analysis will be performed using SAS software version 9.4

13. STUDY MONITORING

Apart from the blood samples taken specifically for the study, this research does not alter the participants’ medical care. We have chosen not to establish an independent data safety monitoring board for this study.

14. ACCESS TO DATA AND SOURCE DOCUMENTS

14.1. Access to Data

The sponsor is responsible for obtaining agreement from all parties involved in the study to guarantee direct access to all study sites, source data, source documents, and reports for quality control and audit purposes by the sponsor. Investigators will make available all documents and individual data strictly necessary for monitoring, quality control, and audit of the research to persons with access authorization, in accordance with current legislative and regulatory provisions (Articles L.1121-3 and R.5121-13 of the Public Health Code)

Access to the electronic case report form (e-CRF) will be granted to investigators or clinical research staff (CRAs/technicians) identified in the protocol or delegation log. Each user will have an account in the data capture system with role-based access corresponding to their function. These roles ensure specific access rights. Each user will receive a username and a unique password by email, and passwords will be 8 characters and changed regularly

14.2. Source Data

Any original document or object that proves the existence or accuracy of a data point or fact recorded during the study is considered a source document. Source documents include the patient’s medical records containing the clinical exam notes, any paraclinical exam results (if required), the operative report, postoperative clinical follow-up notes, as well as the data collection sheet filled out in the operating room by the anesthesiologist (this sheet will be filed in the patient’s chart).

14.3. Data Confidentiality

In compliance with current legal provisions (Articles L.1121-3 and R.5121-13 of the Public Health Code), persons with direct access to source data must take all precautions to ensure the confidentiality of information relating to the investigational medicinal products, the research, and the individuals participating in it—particularly regarding the participants’ identities and the study results. These individuals, as well as the investigators themselves, are bound by professional secrecy.

During and after the research, the data collected on participants and transmitted to the sponsor by the investigators (or other specialized collaborators) will be anonymized. Under no circumstances should the names of participating individuals or their addresses be revealed; identifying information will be coded.

Data confidentiality will be maintained by using a coding system for patient information, composed of: the patient’s initials (first letter of last name and first letter of first name(s)) and a 5-digit code number (a two-digit center number followed by a three-digit patient inclusion number from 001 to 999 in chronological order of inclusion)

The sponsor will ensure that each study participant has given written consent for access to their individual data that are strictly necessary for study monitoring, quality control, and audit, as per the legislative and regulatory requirements in force.

15. QUALITY CONTROL AND ASSURANCE

15.1. Instructions for Data Collection

All information required by the protocol must be recorded as it is obtained in the electronic case report form (CRF). Once data entry for a page is completed and data are final, the investigator or person entering the data should mark the page as complete (“lock” the page) so that the data manager can perform data checks for consistency. If a data value cannot be obtained, a specific missing data code (NA or A for not applicable, ND or D for not done, NK or K for not known) should be used to indicate that the data was not collected and cannot be retrieved later (e.g., an assessment done only for research purposes that was not documented in the medical record).

If an entire page of the CRF has all data missing, the investigator or data entry person should mark that page as “Not applicable” in the e-CRF. All access to the study data and any modifications will be logged by the data capture software (Ennov Clinical®).

15.2. Study Monitoring (Site Visits)

Study monitoring will be performed by a clinical research technician. Under the coordination of the lead investigator, this person will be responsible for:

Logistical organization and oversight of the study progress,

Preparing reports on study progress,

Verifying that the CRF is updated (issuing queries for missing information, corrections, etc.),

Handling shipment of blood samples,

Transmitting SAE reports to the sponsor.

The research technician will work according to standardized operating procedures and in collaboration with the clinical research associate (CRA) appointed by the sponsor

15.3. Quality Control

A clinical research associate (CRA) appointed by the sponsor will visit each investigator’s site per a monitoring plan established by the sponsor—at study initiation, periodically during the study (frequency depending on inclusion rate), and at study close-out. During these visits, the following elements will be reviewed: Informed consent documentation, Adherence to the protocol and the defined procedures, Quality of data recorded in the CRF: accuracy, missing data, consistency of data with source documents (medical records, appointment books, original lab results, etc.), Study product management (if applicable).

Each visit will be documented by a monitoring report in writing.

15.4. Data Management

Data collected directly during patient follow-up will be entered once into an electronic case report form by the principal investigator or authorized persons listed on the delegation log, via the web platform (<https://recherche-clinique.chu-amiens.fr/CSOnline/>)

Pre-defined consistency checks in the database will help verify data quality. Queries for correction will be sent to the investigator if inconsistencies are detected

At the end of the study, the database will be locked (frozen) in Ennov Clinical® by a Data Manager from the DRCI of CHU Amiens-Picardie

The centralized data will then be exported and analyzed using SAS® v9.4

The data will subsequently be processed using SAS® v9.4 to perform all statistical analyses as outlined in the “Statistical Aspects” section

15.5. Audit and Inspection

An audit may be conducted at any time by individuals appointed by the sponsor who are independent of the study team. The goal of an audit is to ensure the quality of the study, the validity of its results, and compliance with current laws and regulations

Investigators agree to comply with the sponsor’s requirements and those of regulatory authorities regarding an audit or inspection of the study. An audit can cover all stages of the research, from protocol development to publication of results and archiving of data and documents produced during the study

16. ETHICAL AND REGULATORY CONSIDERATIONS

The sponsor and investigator(s) commit to conducting this research in accordance with the Public Health Code, Good Clinical Practice (ICH GCP E6(R2) of 1996 and the decision of November 24, 2006), and the Declaration of Helsinki (latest version available on the WMA website)

The study will be carried out as described in this protocol. Except in emergency situations requiring specific therapeutic measures, the investigator(s) agree to adhere to the protocol in all respects, particularly concerning informed consent and the notification and follow-up of serious adverse events

CHU Amiens-Picardie, the sponsor of this research, has obtained civil liability insurance for the study with SHAM, in accordance with Article L1121-10 of the Public Health Code

The data recorded during this research will be processed electronically at the Biostatistics Unit of CHU Amiens-Picardie, in compliance with French Data Protection Act No.78-17 of January 6, 1978 (as amended by Law 2004-801 of August 6, 2004) and the EU General Data Protection Regulation (GDPR)

In accordance with the amended Data Protection Act (last modified December 12, 2018) and under GDPR (EU 2016/679) Chapter III “Rights of the Data Subject,” participants have the right to object to data processing, and rights of access, rectification, erasure, and restriction of processing of personal data concerning them

CHU Amiens-Picardie, represented by its current legal representative, is the data controller for this study per GDPR (since it determines the purposes and means of the data processing in this study). Article 9 of the GDPR allows the processing of special categories of personal data, including health data, for research purposes

Participants can exercise their data rights through the Data Protection Officer (DPO) of CHU Amiens-Picardie (email: dpo@chu-amiens.fr)

For any complaints regarding the processing of health data, participants may contact the French Data Protection Authority (CNIL) via the website: <https://www.cnil.fr/fr/webform/adresser-une-plainte:content>.

This research falls under the “Reference Methodology” MR-001 pursuant to Article 54, paragraph 5 of the amended Data Protection Act of 6 January 1978. This framework was approved by a decision on January 5, 2006. CHU Amiens-Picardie has signed a compliance commitment to this Reference Methodology.

Protocol Amendments: Any substantial modification—i.e., any change that may significantly affect the protection of participants, the study’s validity, the study results, the quality and safety of the investigational products, the interpretation of supporting scientific documents, or the conduct of the study—must be described in a written amendment submitted to the sponsor. The amendment must obtain a favorable opinion from the CPP and authorization from the ANSM before it can be implemented

Non-substantial modifications (ones without significant impact on any aspect of the study) will be communicated to the CPP for information purposes. All amendments will be approved by the sponsor and by all relevant parties in the study before submission to CPP and ANSM. This approval may require a meeting of the Scientific Committee and/or Safety Committee (if such committees are constituted for the study)

All protocol amendments must be brought to the attention of all investigators participating in the research. The investigators agree to comply with the content of any approved amendments

Any amendment that changes patient management or the benefits, risks, or constraints of the research will require a new patient information sheet and consent form, which must be collected using the same procedure as the initial consent.

17. ARCHIVING OF STUDY DOCUMENTS AND DATA

The following documents related to the study will be archived in compliance with Good Clinical Practice:

By the Investigators:

– For 15 years after study completion (for studies involving drugs, medical devices, in vitro diagnostics, or other regulated products):

• The protocol and any protocol amendments.

• Case report forms (copies).

• Source data files for participants who signed consent.

• All other documents and correspondence related to the study. – For 30 years after study completion:

• The original signed informed consent forms of participants. All these documents remain under the investigator’s responsibility for the regulatory archiving duration

By the Sponsor:

– For 15 years after study completion (for studies involving drugs, medical devices, in vitro diagnostics, or other regulated products):

• The protocol and any amendments.

• The original case report forms.

• All other documents and correspondence related to the study. – For 30 years after study completion:

• A copy of the signed informed consent forms of participants.

• Documents related to serious adverse events. All these documents remain under the sponsor’s responsibility for the regulatory archiving duration

No relocation or destruction of study archives may occur without the sponsor’s agreement. At the end of the regulatory archiving period, the sponsor will be consulted regarding the destruction of records. All data, documents, and reports may be subject to audit or inspection at any time

18. PUBLICATION RULES

18.1. Scientific Communications

Data analysis will be performed by the Biostatistics Unit of CHU Amiens-Picardie, resulting in a written report that will be submitted to the sponsor, who will in turn transmit it to the CPP and the regulatory authority as required. Any written or oral communication of the study results must be approved by the coordinating investigator and, if applicable, by any committee established for the study. The publication of primary results will include the name of the sponsor, the names of all investigators who enrolled or followed patients in the study, as well as the names of methodologists, biostatisticians, and data managers who contributed, and members of any study committee. International standards for authorship and publication (ICMJE Uniform Requirements for Manuscripts, April 2010) will be followed.

18.2. Communication of Results to Patients

In accordance with French Law No.2002-303 of March 4, 2002, patients are entitled to be informed, upon request, of the overall results of the research. The global results of the study will be made available to participants who desire them.

18.3. Data Transfer

Data collection is carried out by the investigators or authorized staff listed on the task delegation log. Data management is performed by a Data Manager in the DRCI of CHU Amiens-Picardie. No transfer of data is planned for this protocol. However, in the event of any data transfer, the conditions of transfer of all or part of the study database will be decided by the sponsor and will be subject to a written agreement.

19. REFERENCES

Alfonsi P., Slim K., Chauvin M., Mariani P., Faucheron J.-L., Fletcher D., et al. (Working group of the French Society of Anesthesia and Intensive Care (SFAR) and the French Society of Digestive Surgery (SFCD)). Guidelines for enhanced recovery after elective colorectal surgery (SFCD). Journal of Visceral Surgery, 2014 (citation details not fully provided in source).

French Society of Anesthesia and Intensive Care (SFAR). Perimedullary blocks in adults. Annales Françaises d’Anesthésie et de Réanimation, 26 (2007): 720–752.

Beaussier M., Delbos A., Maurice-Szamburski A., Ecoffey C., & Mercadal L. (2018). Perioperative use of intravenous lidocaine. Drugs, 78(12): 1229–1246.

Weinberg L., Peake B., Tan C., Nikfarjam M. (2015). Pharmacokinetics and pharmacodynamics of lignocaine: A review. World J Anesthesiol, 4(2): 17–29.

Aubrun F., Nouette-Gaulain K., Fletcher D., Belbachir A., Beloeil H., Carles M., et al. Update of the guideline on postoperative pain management, SFAR. (Société Française d’Anesthésie et de Réanimation, 2016).

Knudsen K., Beckman Suurküla M., Blomberg S., Sjövall J., Edvardsson N. (1997). Central nervous and cardiovascular effects of i.v. infusions of ropivacaine, bupivacaine and placebo in volunteers. Br. J. Anaesth., 78(5): 507–514.

Perotti L., Cusato M., Ingelmo P., Niebel T.L., Somaini M., Riva F., et al. (2015). Differences between the systemic pharmacokinetics of levobupivacaine and ropivacaine during continuous epidural infusion: A randomized, double-blind trial. Anesth. Analg., 121(2): 348–356.

French Public Drug Database. Ropivacaine – Summary of Product Characteristics. (Accessible via <http://base-donnees-publique.medicaments.gouv.fr>).

Koppert W., Weigand M., Neumann F., Sittl R., Schüttler J., Schmelz M., Hering W. (2004). Perioperative intravenous lidocaine has preventive effects on postoperative pain and morphine consumption after major abdominal surgery. Anesth. Analg., 98(4): 1050–1055.

Herroeder S., Pecher S., Schönherr M.E., Kaulitz G., Hahnenkamp K., Friess H., et al. (2007). Systemic lidocaine shortens length of hospital stay after colorectal surgery: a double-blinded, randomized, placebo-controlled trial. Ann. Surg., 246: 192–200.

Bryson G.L., Charapov I., Krolczyk G., Taljaard M., Reid D. (2010). Intravenous lidocaine does not reduce length of hospital stay following abdominal hysterectomy. Can. J. Anaesth., 57: 759–766.
